# USP5 Stabilizes TGFBR1 to Drive Vascular Smooth Muscle Cell Senescence and Atherosclerosis

**DOI:** 10.1002/advs.76911

**Published:** 2026-08-05

**Authors:** Xinhai Cui, Yuanlong Hu, Lin Lin, Lei Zhang, Chao Li, Yunlun Li

**Affiliations:** ^1^ College of Traditional Chinese Medicine Shandong University of Traditional Chinese Medicine Jinan China; ^2^ Innovation Research Institute of Traditional Chinese Medicine Shandong University of Traditional Chinese Medicine Jinan China; ^3^ Department of Cardiovascular Affiliated Hospital of Shandong University of Traditional Chinese Medicine Jinan China

**Keywords:** atherosclerosis, glycolysis, senescent vascular smooth muscle cell, TGFBR1, USP5

## Abstract

Vascular smooth muscle cell (VSMC) senescence contributes importantly to atherosclerotic plaque progression, yet the upstream mechanisms remain incompletely understood. Here, by integrating single‐cell RNA sequencing analysis with human plaque validation, we found that TGFBR1 is enriched in senescent VSMCs in atherosclerotic lesions and correlates with senescence‐associated markers. In vivo, VSMC‐specific TGFBR1 knockin in Apoe‐deficient mice accelerated plaque development and increased VSMC senescence. Mechanistically, we identified the deubiquitinase USP5 as a previously unrecognized stabilizer of TGFBR1. USP5 directly interacted with TGFBR1 and selectively removed K48‐linked polyubiquitin chains at lysine 213, thereby preventing proteasomal degradation. Stabilized TGFBR1 suppressed the mitochondrial enzyme IDH2, driving a metabolic shift toward glycolysis that sustained apoptosis resistance in senescent VSMCs. Conversely, VSMC‐specific knockdown of USP5 reduced TGFBR1 expression, restored IDH2 expression, attenuated glycolytic remodeling, and mitigated atherosclerosis *in viv*o. Our findings reveal a USP5‐TGFBR1‐IDH2 axis in which site‐specific deubiquitination at K213 links receptor stability to metabolic remodeling and VSMC senescence, identifying USP5 as a potential therapeutic target for atherosclerosis.

## Graphical abstract

This study reveals that USP5 drives vascular smooth muscle cell senescence and atherosclerosis by stabilizing TGFBR1, suppressing IDH2, and promoting glycolytic reprogramming, identifying the USP5‐TGFBR1‐IDH2 axis as a potential therapeutic target.

## 1 Introduction

Atherosclerosis is a chronic, progressive, age‐related immunoinflammatory disease driven by lipids [1]. Although therapeutic approaches have advanced, atherosclerosis remains a leading cause of mortality worldwide, underscoring the need to identify novel therapeutic targets to combat this devastating condition [2]. Recently, emerging evidence indicates that lipid abnormalities not only promote the transformation of macrophages into foam cells but also exert pro‐atherosclerosis effects by inducing senescence in vascular cells [3, 4]. The senescent vascular smooth muscle cells (VSMCs) are the predominant cell type present in atherosclerotic plaques [5]. The hydrolytic products of modified low‐density lipoprotein cholesterol and remnant cholesterol can induce senescence in VSMCs, leading to stress‐induced premature senescence (SIPS) [6]. Senescent VSMCs accumulate within atherosclerotic plaques and shape a pro‐atherosclerotic microenvironment through their senescence‐associated secretory phenotype (SASP), thereby driving pathological progression [7]. Moreover, senescent VSMCs exhibit reduced collagen secretion, which leads to decreased synthesis of the extracellular matrix and compromises plaque stability [8]. However, the molecular mechanisms governing VSMC senescence during atherosclerosis are incompletely understood.

Transforming growth factor‐β (TGF‐β) signaling plays complex and context‐dependent roles in vascular biology and atherosclerosis [9, 10]. Emerging evidence indicates that aberrant activation of this pathway can promote vascular remodeling [11]. Central to this signaling cascade is transforming growth factor‐β receptor 1 (TGFBR1), the key kinase receptor that mediates downstream SMAD‐dependent transcriptional programs and can also engage non‐canonical signaling pathways [12, 13]. Within the atherosclerotic microenvironment, hyperactive TGFBR1 signaling has been shown to exacerbate the osteogenic transdifferentiation of VSMCs, thereby accelerating vascular calcification [14]. Moreover, sustained TGF‐β activation is a recognized inducer of cellular senescence, operating via the upregulation of cyclin‐dependent kinase inhibitors such as p21 and p15 [15, 16]. However, whether dysregulation of TGF‐β signaling at the receptor level, particularly through aberrant activation of TGFBR1, directly drives VSMC senescence during atherosclerosis progression remains unclear. Importantly, the pathogenic consequences of this cascade are driven not merely by ligand availability, but by the precise post‐translational regulation of TGFBR1 protein stability [17]. Under physiological conditions, TGFBR1 is highly susceptible to recruitment and degradation via the SMAD7‐SMURF2 complex‐mediated ubiquitination pathway, a process essential for maintaining appropriate signal termination [18]. However, recent oncological studies have revealed that specific deubiquitinating enzymes (DUBs) can effectively suppress this proteolytic degradation to stabilize TGFBR1, albeit through distinct modes of action. For instance, USP15 does not bind to the receptor directly; instead, it is recruited to the SMAD7‐E3 ligase complex, where it reverse TGFBR1 ubiquitination and amplify downstream signaling to promote development of glioblastoma [19, 20]. Conversely, USP4 directly interacts with and deubiquitinates TGFBR1 in a SMAD7‐independent manner, enabling the persistent accumulation of the receptor at the plasma membrane to drive breast cancer progression [21]. While the DUB‐mediated post‐translational stabilization of TGFBR1 is well‐established in various cancers, the specific deubiquitinase responsible for stabilizing TGFBR1 to drive the senescent phenotype in VSMCs during atherosclerosis remains unclear.

In this study, we investigated the role of TGFBR1 in VSMC senescence during atherosclerosis progression. We observed that TGFBR1 is enriched in senescent VSMCs within atherosclerotic lesions and that VSMC‐specific TGFBR1 activation accelerates plaque development in vivo. Mechanistically, sustained TGFBR1 signaling was associated with suppression of the mitochondrial enzyme IDH2 and a shift toward enhanced glycolysis, which contribute to apoptosis resistance and maintenance of the senescent phenotype. Importantly, we further identified the deubiquitinase USP5 as an upstream regulator of TGFBR1 stability that directly removes K48‐linked polyubiquitin chains from TGFBR1 at lysine 213. These findings support a USP5‐TGFBR1‐IDH2 regulatory axis that sustains VSMC senescence, exposing a promising therapeutic target for mitigating atherosclerosis.

## 2 Results

### 2.1 TGFBR1 is Upregulated in Senescent VSMCs During Atherosclerosis

To elucidate the transcriptomic alterations associated with atherosclerosis, we re‐analyzed single‐cell RNA sequencing (scRNA‐seq) data from whole‐aorta samples of mice (GEO dataset GSE239591). Unsupervised clustering identified eight distinct major cell populations within the aortic landscape (Figure 1A). Notably, violin plot analysis revealed that the upregulation of *Tgfbr1* expression under a high‐fat diet (HFD) conditions was most significant (*p* < 0.0001) in VSMCs (Figure 1B). Furthermore, UMAP feature plots demonstrated a co‐expression of *Tgfbr1* with the senescence marker *Cdkn1a* (encoding p21) specifically within the VSMC cluster derived from atherosclerotic aortas (Figure 1C,D). These scRNA‐seq findings prompted the hypothesis that TGFBR1 is linked to VSMC senescence during atherosclerosis. To validate this clinical relevance, we evaluated human carotid artery specimens. Immunofluorescence staining revealed that VSMCs within atherosclerotic plaques displayed prominent senescent features, accompanied by a markedly higher co‐localization of TGFBR1 and p21 compared to VSMCs in adjacent disease‐free regions (Figure 1E). Similarly, in vivo assessment using *Apo* *e* ^−/−^ mice demonstrated that the relative fluorescence intensity of TGFBR1, as well as its co‐localization with α‐SMA^+^/p21^+^ senescent VSMCs, was significantly augmented in the aortic root plaques of HFD‐fed mice compared to the normal chow diet (NC) group (Figure 1F). This progressive accumulation of TGFBR1 during atherosclerosis was further corroborated by Western blot analysis of whole‐aorta lysates, which showed a concurrent elevation of TGFBR1, p21, and p16 proteins (Figure 1G). To investigate this regulatory mechanism in vitro, we evaluated TGFBR1 expression in cultured VSMCs across three distinct states: active proliferation (Ctrl), replicative senescence (RS), and SIPS. Compared to the Ctrl, both the RS and SIPS models exhibited robust senescent phenotypes, characterized by an increased percentage of SA‐β‐gal‐positive cells (Figure 1H) and elevated protein levels of TGFBR1 and classical senescence markers (Figure 1I). Cell‐cell communication analysis of the scRNA‐seq data further suggested enhanced ligand‐receptor interactions between senescent VSMCs and monocytes/macrophages, including Il6‐Il6r, Ccl2‐Ccr2, Cxcl12‐Cxcr4, Mif‐Cd74_Cxcr4/Cd44, and Spp1‐Cd44 (Figure S1). Consistent with this prediction, RS and SIPS‐induced senescent VSMCs showed a pronounced upregulation of SASP factors (Figure 1J and Table S1). Taken together, these data indicate that TGFBR1 is associated with VSMC senescence during atherosclerosis.

**FIGURE 1:**
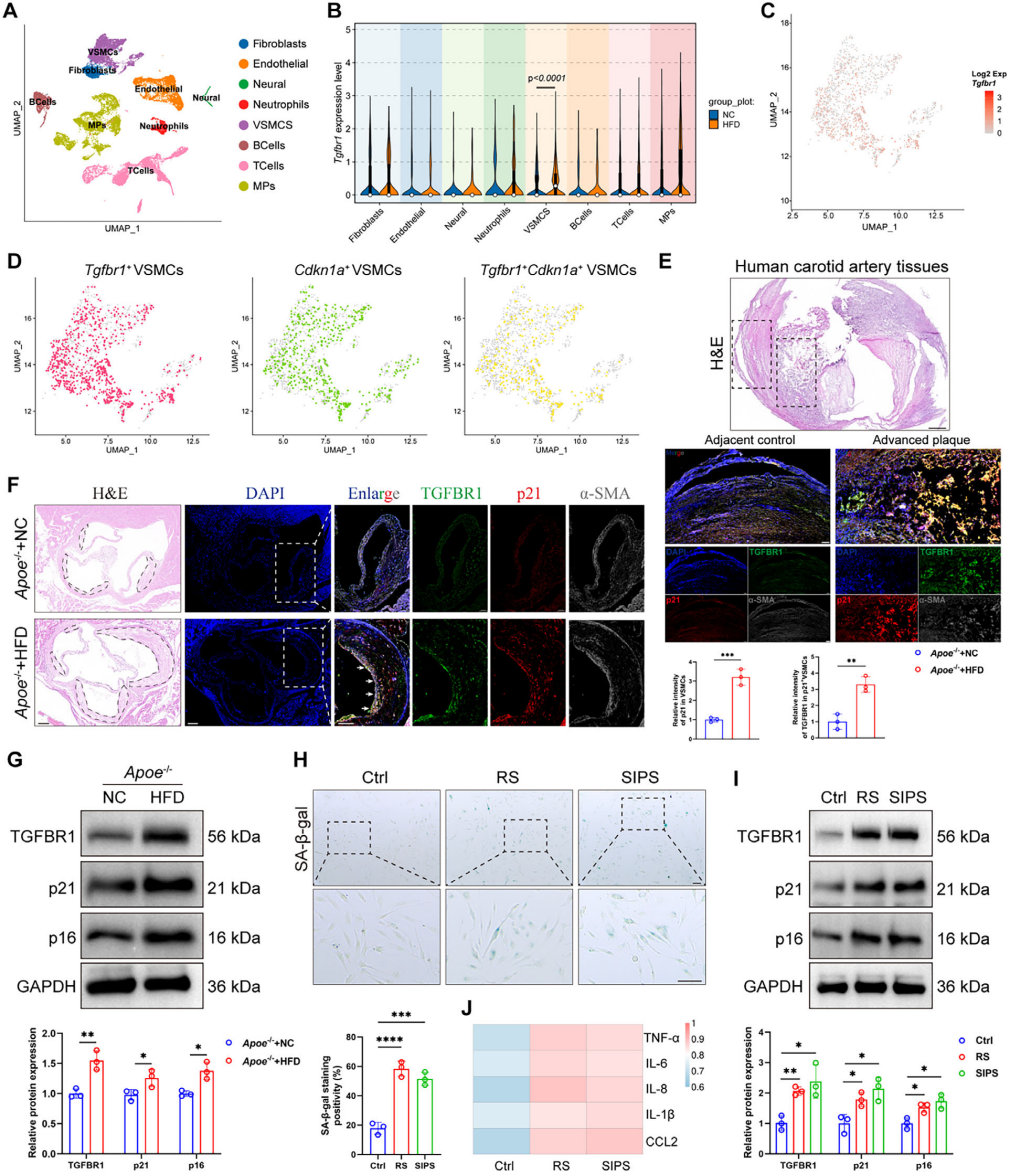
Upregulation of TGFBR1 is associated with VSMC senescence in atherosclerosis. (A) UMAP plot of eight major cell clusters identified from atherosclerotic aortas via single‐cell RNA sequencing. (B) Expression levels of *Tgfbr1* across different cell types. (C) Feature plot showing the distribution of *Tgfbr1* in VSMCs. (D) Cell clusters expressing *Tgfbr1* (*Tgfbr1* ^+^ VSMCs), *Cdkn1a* (*Cdkn1a* ^+^ VSMCs), and both (*Tgfbr1* ^+^ *Cdkn1a* ^+^ VSMCs). (E) Representative images of haematoxylin and eosin (H&E) staining and immunofluorescence staining of TGFBR1 (green), p21 (red), and α‐SMA (grey) in human carotid artery plaques and adjacent non‐plaque regions (*n* = 3). Scale bar: 100 µm. (F) Representative images of H&E staining and immunofluorescence staining of TGFBR1 (green), p21 (red), and α‐SMA (grey) in aortic root from *Apoe* ^−/−^ mice fed normal chow diet (NC) and high‐fat diet (HFD) for 12 weeks. The lesion areas are outlined by dashed lines. Quantitative analysis of relative fluorescence intensity and colocalization of TGFBR1, p21, and α‐SMA (*n* = 3). Scale bar: 100 µm. (G) Representative Western blot images and quantitative analysis of TGFBR1, p21, and p16 expression in mouse aortas (*n* = 3). (H) SA‐β‐gal staining of cultured VSMCs undergoing active proliferation (Ctrl), replicative senescence (RS), or stress‐induced premature senescence (SIPS), with quantitative analysis (*n* = 3). Scale bar: 100 µm. (I) Representative Western blot images and quantitative analysis of TGFBR1, p21, and p16 expression in VSMCs in three distinct states: Ctrl, RS, and SIPS (*n* = 3). (J) Heatmap showing the expression of SASP in Ctrl, RS, and SIPS VSMCs (*n* = 6). Data are presented as mean ± SD and were analyzed by Wilcoxon rank‐sum test (B), two‐tailed unpaired Student's t‐test (F, G), or one‐way ANOVA followed by Tukey's multiple‐comparisons test (H, I). ^*^ *p*  <  0.05, ^**^ *p*  <  0.01, ^***^ *p*  <  0.001, ^****^ *p*  <  0.0001.

### 2.2 VSMC‐Specific TGFBR1 Knockin Exacerbates Atherosclerosis and Promotes VSMC Senescence

To investigate the in vivo role of TGFBR1 in VSMCs, we generated VSMC‐specific TGFBR1 knockin mice with *Apoe* ^KO^ and fed them a HFD for 12 weeks. Successful knockin efficiency was verified via Western blot, which revealed significantly upregulated TGFBR1 protein expression in VSMC‐containing tissues, including the aorta, heart, and lung, of *Apoe* ^KO^ *Tgfbr1* ^SMCKI^ mice compared to *Apoe* ^KO^ *Tgfbr1* ^WT^ controls (Figure 2A). Notably, metabolic parameters, including body weight and serum lipid profiles, exhibited no significant differences between the two groups (Figure 2B), suggesting that the exacerbated vascular phenotype is driven by local VSMC alterations rather than systemic lipid metabolism. Morphological assessment of the whole aorta using en face Oil Red O staining demonstrated a significantly exacerbated atherosclerotic plaque burden in HFD‐fed *Apoe* ^KO^ *Tgfbr1* ^SMCKI^ mice (Figure 2C). Consistently, histological cross‐sections of the aortic root revealed larger necrotic core areas (Figure 2D), reduced collagen deposition (Figure 2E), increased CD68^+^ macrophage infiltration (Figure 2F), and enhanced intra‐plaque neutral lipid accumulation (Figure 2G). These data indicate that VSMC‐specific TGFBR1 knockin accelerates advanced plaque formation and instability. We next examined whether TGFBR1 overexpression impacted VSMC senescence in vivo. Immunofluorescence staining of the aortic root revealed that the targeted knockin resulted in a marked elevation of TGFBR1 and the senescence marker p21, specifically within α‐SMA^+^ VSMCs residing in the plaques (Figure 2H). This in vivo pro‐senescent shift was further corroborated by significantly intensified SA‐β‐gal staining in isolated aortas (Figure 2I) and elevated protein expression of TGFBR1, p21, and p16 in whole‐aorta tissue (Figure 2J). Collectively, these results demonstrate that elevated TGFBR1 expression promotes VSMC senescence and is associated with aggravated atherosclerotic development.

**FIGURE 2:**
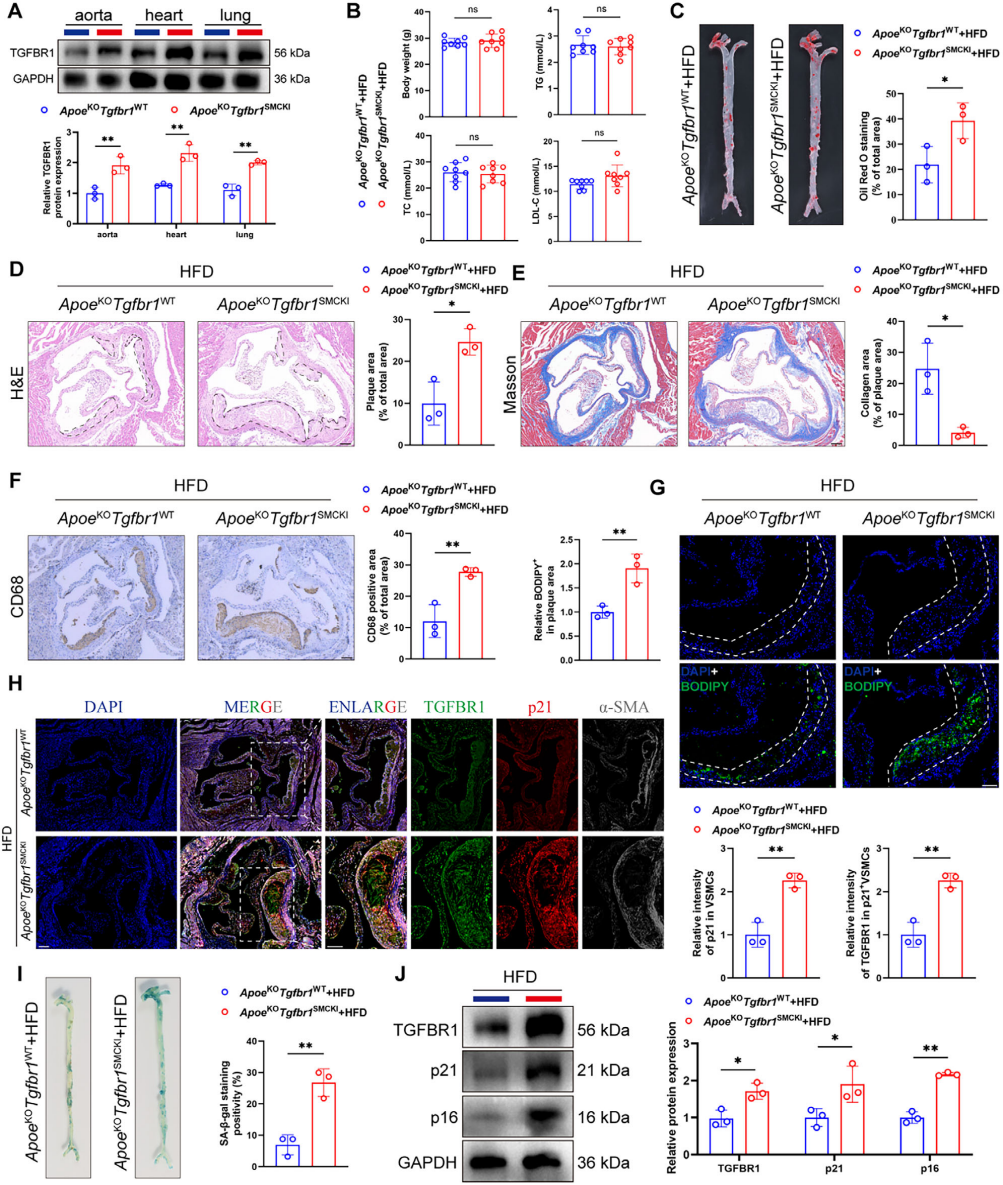
VSMC‐specific TGFBR1 knockin accelerates atherosclerosis and VSMC senescence. (A) Representative Western blot images and quantitative analysis of TGFBR1 expression in aorta, heart, and lung tissues from *Apoe* ^KO^ *Tgfbr1* ^WT^ and *Apoe* ^KO^ *Tgfbr1* ^SMCKI^ mice (*n* = 3). (B) Body weight and serum levels of triglycerides (TG), total cholesterol (TC), and low‐density lipoprotein cholesterol (LDL‐C) in mice fed a HFD for 12 weeks (*n* = 8). (C) Representative images of aortas stained with Oil Red O from HFD‐fed mice, accompanied by quantitative analysis of the total lesion area (*n* = 3). (D–G) Representative cross‐sections of aortic root stained with H&E, Masson, CD68, and immunofluorescence staining for BODIPY (*n* = 3). Scale bar: 100 µm. The lesion areas are outlined by dashed lines. Accompanying bar graphs display the quantitative analyses of plaque area, collagen proportion, CD68‐positive area, and relative BODIPY fluorescence intensity within the lesions. (H) Representative immunofluorescence images and quantitative analysis demonstrating the expression and colocalization of TGFBR1 (green), p21 (red), and α‐SMA (grey) in aortic root plaques (*n* = 3). Scale bar: 100 µm. (I) SA‐β‐gal staining of isolated mouse aortas with corresponding quantitative positivity rates (*n* = 3). (J) Representative Western blot images and quantitative analysis of TGFBR1, p21, and p16 expression in mouse aortas (*n* = 3). Data are presented as mean ± SD and were analyzed by two‐tailed unpaired Student's t‐test (A–J). ^*^ *p*  <  0.05, ^**^ *p*  <  0.01, ns: *p*  >  0.05.

### 2.3 TGFBR1 Overexpression Induces an Anti‐apoptotic Phenotype to Sustain VSMC Senescence

Senescent cells characteristically develop a profound resistance to apoptosis, a survival mechanism closely associated with the upregulation of anti‐apoptotic proteins such as BCL‐2 [22]. To determine whether the pro‐senescent effect of TGFBR1 depends on this anti‐apoptotic phenotype, we first evaluated in vivo plaques. Immunofluorescence staining of aortic roots revealed that BCL‐2 expression specifically within senescent (p21^+^) VSMCs was significantly augmented in *Apoe* ^KO^ *Tgfbr1* ^SMCKI^ mice compared to *Apoe* ^KO^ *Tgfbr1* ^WT^ mice (Figure 3A). Consistent with this, Western blot analysis of aortas demonstrated a markedly elevated BCL‐2/BAX ratio in the VSMC‐specific TGFBR1 knockin mice (Figure 3B), indicating an enhanced anti‐apoptotic capacity in vivo. To validate this mechanistically, we transfected a TGFBR1 expression plasmid into VSMCs undergoing SIPS. Assessment of the mitochondrial membrane potential using JC‐1 dye revealed that TGFBR1‐overexpressing VSMCs maintained robust mitochondrial integrity, as indicated by intense red J‐aggregate fluorescence (Figure 3C). Furthermore, TGFBR1 overexpression significantly upregulated BCL‐2 expression and the BCL‐2/BAX ratio compared to the vector control, which was confirmed by both Western blot (Figure 3D) and immunofluorescence (Figure 3E). Driven by this enhanced survival capacity, these TGFBR1‐overexpressing VSMCs exhibited exacerbated senescent features, including an increased proportion of SA‐β‐gal positive cells (Figure 3F), upregulation of the SASP (Figure 3G and Table S2), and elevated expression of the senescence markers p21 and p16 (Figure 3H). Notably, disruption of this survival state by TGFBR1 knockdown in senescent VSMCs significantly promoted cellular apoptosis, as evidenced by Annexin V‐FITC staining (Figure 3I). Consequently, the clearance of these senescent cells led to a marked reduction in the overall SA‐β‐gal positivity (Figure 3J). These findings indicate that elevated TGFBR1 expression sustains the senescent state of VSMCs by promoting an anti‐apoptotic phenotype.

**FIGURE 3:**
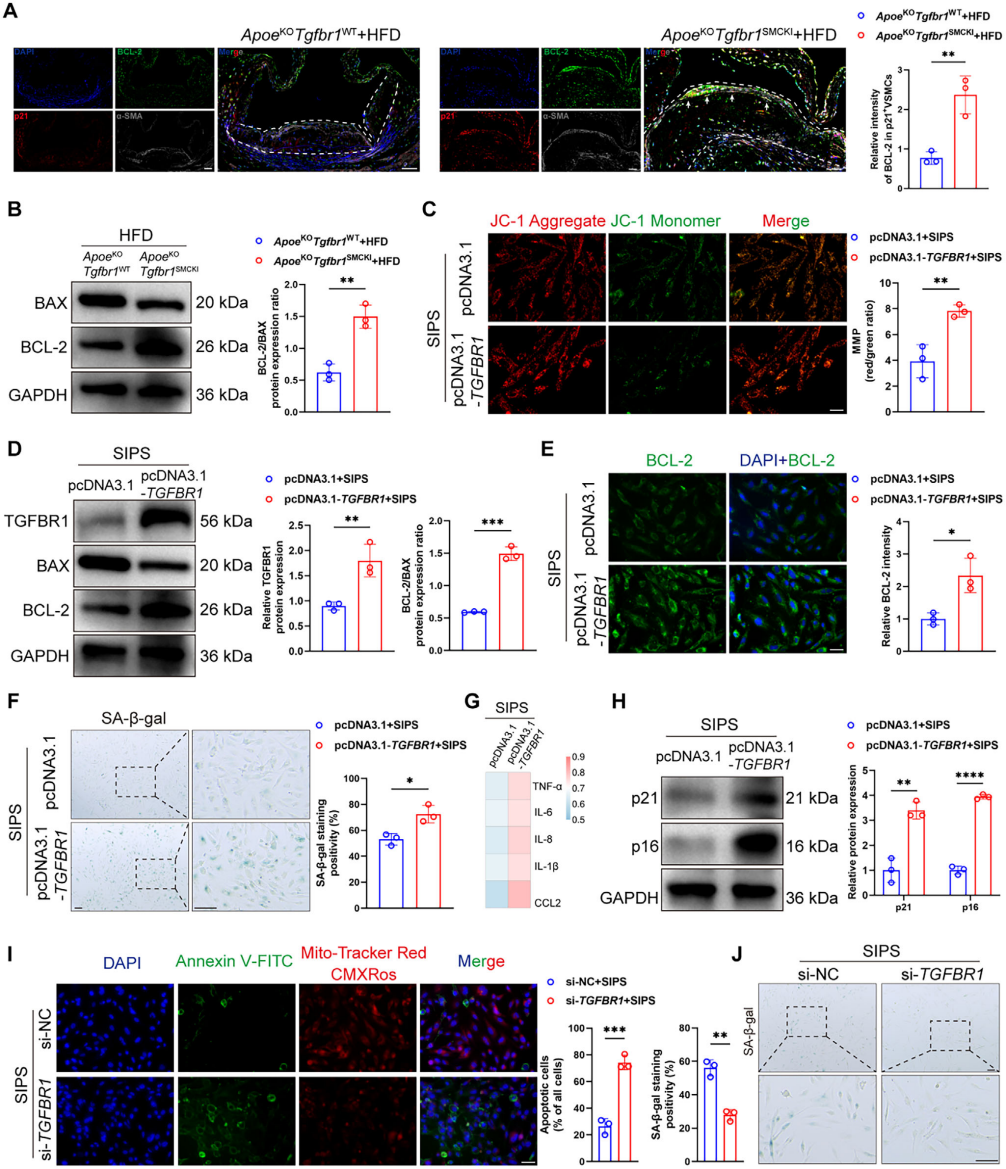
TGFBR1 sustains VSMC senescence by promoting an anti‐apoptotic phenotype. (A) Representative immunofluorescence images and quantitative analysis of BCL‐2 (green), p21 (red), and α‐SMA (grey) colocalization in the aortic roots of HFD‐fed *Apoe* ^KO^ *Tgfbr1* ^WT^ and *Apoe* ^KO^ *Tgfbr1* ^SMCKI^ mice (n = 3). Scale bar: 50 µm. (B) Representative Western blot images and quantitative analysis of BCL‐2 and BAX expression in mouse aortas (*n* = 3). (C) Assessment of mitochondrial membrane potential (MMP) using JC‐1 staining in VSMCs undergoing SIPS, transfected with an empty vector (pcDNA3.1) or a TGFBR1 overexpression plasmid (pcDNA3.1‐*TGFBR*1). The bar graph shows the ratio of J‐aggregates (red) to J‐monomers (green) (*n* = 3). Scale bars: 50 µm. (D) Representative Western blot images and quantitative analysis of BCL‐2 and BAX expression in TGFBR1‐overexpressing senescent VSMCs (*n* = 3). (E) Representative immunofluorescence staining and quantification of BCL‐2 (green) in VSMCs in a SIPS state following TGFBR1 overexpression (*n* = 3). Scale bar: 50 µm. (F) SA‐β‐gal staining and quantitative positivity rates in the indicated VSMC groups (*n* = 3). Scale bar: 100 µm. (G) Heatmap showing the relative expression of SASP‐related factors in TGFBR1‐overexpressing senescent VSMCs (*n* = 6). (H) Representative Western blot images and quantitative analysis of p21 and p16 expression in TGFBR1‐overexpressing senescent VSMCs (*n* = 3). (I) Representative fluorescence images and quantitative analysis of apoptotic cells (Annexin V‐FITC, green) and mitochondrial tracking (Mito‐Tracker Red CMXRos, red) in VSMCs undergoing SIPS following TGFBR1 knockdown (si‐*TGFBR*1) vs. negative control (si‐NC) (*n* = 3). Scale bars: 50 µm. (J) SA‐β‐gal staining and quantification of VSMCs in a SIPS state after transfection with si‐NC or si‐*TGFBR1* (*n* = 3). Scale bars: 100 µm. Data are presented as mean ± SD and were analyzed by two‐tailed unpaired Student's t‐test (A–F, H–J). ^*^ *p*  <  0.05, ^**^ *p*  <  0.01, ^***^ *p*  <  0.001, ^****^ *p*  <  0.0001.

### 2.4 TGFBR1 Enhances Glycolytic Activity via the Downregulation of IDH2

To delineate the downstream effector network by which TGFBR1 maintains the anti‐apoptotic phenotype in senescent VSMCs, we performed quantitative proteomic analysis on aortic tissues from HFD‐fed *Apoe* ^KO^ *Tgfbr1* ^WT^ and *Apoe* ^KO^ *Tgfbr1* ^SMCKI^ mice. Principal component analysis (PCA) revealed a distinct separation of protein expression profiles between the two groups, indicating substantial proteomic reprogramming driven by VSMC‐specific TGFBR1 overexpression (Figure 4A). GO enrichment analysis highlighted that biological processes such as “regulation of glycogen metabolic process” were significantly altered (Figure 4B). Furthermore, KEGG pathway enrichment identified “Metabolic pathways” and “Cellular senescence” as top‐enriched signaling networks, with “2‐Oxocarboxylic acid metabolism” being particularly prominent (Figure 4C). This metabolic shift was corroborated by GSEA, which demonstrated a significant enrichment of both the 2‐oxocarboxylic acid metabolism and the pro‐senescent p53 signaling pathways in the knockin mice (Figure 4D,E). Among the DEPs within the 2‐oxocarboxylic acid metabolic network, the mitochondrial enzyme IDH2 was markedly downregulated (Figure 4F). Previous studies have shown that IDH2 deficiency can rewire cellular metabolism toward increased glycolytic dependence under stress conditions [23]. Accordingly, we considered IDH2 to be a potential target linking TGFBR1 activation to metabolic remodeling in senescent VSMCs. To validate these proteomic findings, we evaluated IDH2 expression in vivo. Immunofluorescence staining of aortic roots confirmed that IDH2 expression was markedly decreased in the plaque VSMCs of *Apoe* ^KO^ *Tgfbr1* ^SMCKI^ mice compared to *Apoe* ^KO^ *Tgfbr1* ^WT^ mice (Figure 4G). Consistently, in vitro confocal imaging revealed a marked reduction in IDH2 fluorescence intensity within the mitochondria of TGFBR1‐overexpressing VSMCs in a SIPS state (Figure 4H). To determine whether this molecular target was associated with metabolic reprogramming, we conducted real‐time cellular metabolic analyses using a Seahorse XFe96 Analyzer. The results demonstrated that TGFBR1 overexpression significantly elevated both the basal and compensatory glycolytic proton efflux rates (glycoPER) in VSMCs undergoing SIPS (Figure 4I), indicating increased glycolytic flux. This was further substantiated by increased intracellular G6P levels, a key glycolytic intermediate, together with elevated lactate levels (Figure 4J). Conversely, TGFBR1 deficiency in VSMCs undergoing SIPS successfully restored IDH2 expression (Figure 4K) and suppressed the aberrant G6P and lactate overproduction (Figure 4L). We next examined whether canonical TGF‐β/SMAD signaling was involved in TGFBR1‐mediated IDH2 suppression. Senescent VSMCs showed increased TGFBR1 expression and SMAD2/3 phosphorylation compared with Ctrl cells (Figure S2A). However, SMAD3 knockdown failed to restore IDH2 expression or reverse the increase in senescence markers induced by TGFBR1 overexpression (Figure S2B). Together, these results suggest that TGFBR1 suppresses IDH2 through a SMAD‐independent mechanism, thereby promoting glycolytic reprogramming and senescent phenotype of VSMCs.

**FIGURE 4:**
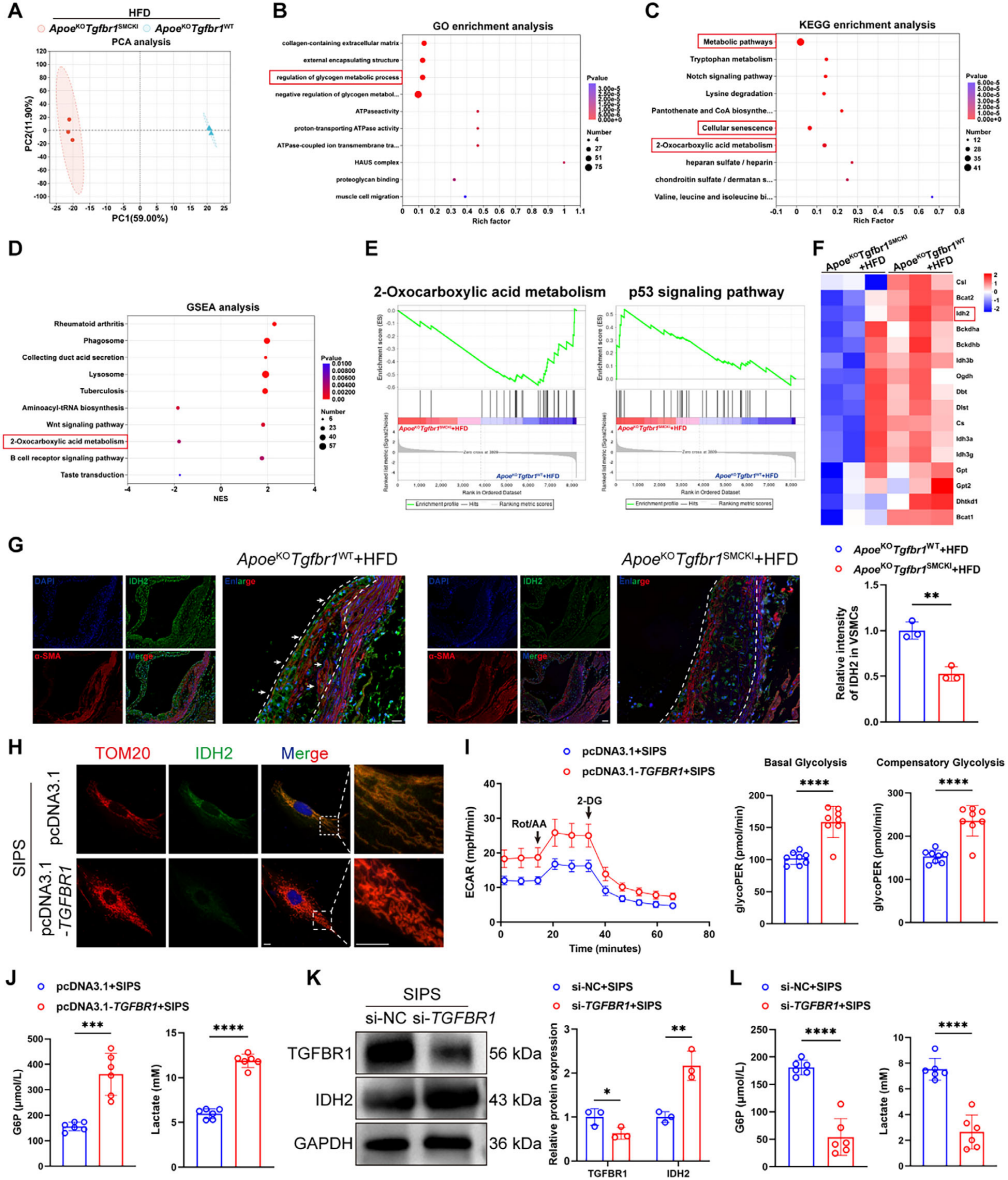
TGFBR1 overexpression downregulates IDH2 expression and drives glycolysis in senescent VSMCs. (A) Principal component analysis (PCA) of proteomic data from aortas of *Apoe* ^KO^ *Tgfbr1* ^WT^ and *Apoe* ^KO^ *Tgfbr1* ^SMCKI^ mice fed a HFD for 12 weeks. GO (B) and KEGG pathway (C) enrichment analyses of the differentially expressed proteins (DEPs) between the two groups. (D,E) Gene Set Enrichment Analysis (GSEA) of the proteins between the two groups. (F) Heatmap analysis of DEPs involved in 2‐Oxocarboxylic acid metabolism. (G) Representative immunofluorescence staining and quantification of IDH2 (green) in α‐SMA^+^VSMCs (red) in the aortic roots of HFD‐fed *Apoe* ^KO^ *Tgfbr1* ^WT^ and *Apoe* ^KO^ *Tgfbr1* ^SMCKI^ mice (*n* = 3). Scale bar: 50 µm. (H) Representative immunofluorescence staining of TOM20 and IDH2 in senescent VSMCs overexpression TGFBR1 (*n* = 3). Scale bar: 10 µm. (I) Real‐time measurement of the extracellular acidification rate (ECAR) using a Seahorse analyzer, with quantitative bar graphs showing basal and compensatory glycolysis in TGFBR1‐overexpressing VSMCs undergoing SIPS (*n* = 8). (J) Glucose‐6‐phosphate (G6P) and lactate levels in VSMCs in a SIPS state following TGFBR1 overexpression (*n* = 6). (K) Representative Western blot images and quantitative analysis of TGFBR1 and IDH2 expression in senescent VSMCs transfected with si‐*TGFBR1* or si‐NC (*n* = 3). (L) G6P and lactate concentrations in VSMCs in a SIPS state following TGFBR1 knockdown (n = 6). Data are presented as mean ± SD and were analyzed by two‐tailed unpaired Student's t‐test (G‐L). ^*^ *p*  <  0.05, ^**^ *p*  <  0.01, ^***^ *p*  <  0.001, ^****^ *p*  <  0.0001.

### 2.5 IDH2 Overexpression Suppresses Glycolytic Activity and Promotes Apoptosis in Senescent VSMCs

To verify whether TGFBR1 promotes apoptosis resistance in senescent VSMCs by downregulating IDH2 expression, IDH2 overexpression plasmid was transfected in VSMCs. We observed an increase in IDH2 expression in the mitochondria of VSMCs undergoing SIPS (Figure 5A). Crucially, overexpression of IDH2 significantly inhibited the glycolytic capacity of senescent VSMCs (Figure 5B). Moreover, the levels of lactate and the intermediate glycolytic metabolite G6P were also reduced (Figure 5C). Deprived of this glycolytic energy supply, VSMCs in a SIPS state lost their anti‐apoptotic phenotype. IDH2 overexpression triggered a sharp increase in the cellular apoptosis rate (Figure 5D) and markedly reduced the BCL‐2/BAX ratio (Figure 5E). To further assess whether TGFBR1 acts upstream of IDH2 in this process, we performed a rescue experiment by stimulating the cells with TGFβ1. Activation of the TGFBR1 pathway partially reversed the metabolic suppression induced by the IDH2 overexpression and restored glycolytic flux (Figure 5F). Consistently, IDH2 overexpression reduced the protein levels of HK2, PFKFB3, and PKM2 in senescent VSMCs, whereas TGFβ1 stimulation partially restored the expression of these glycolytic enzymes (Figure S3). Furthermore, TGFβ1 stimulation preserved the MMP (Figure 5G), and restored the anti‐apoptotic BCL‐2/BAX balance (Figure 5H). Collectively, these reverse‐validation results confirm that TGFBR1‐mediated suppression of IDH2 contributes to maintenance of glycolytic activity and apoptosis resistance in senescent VSMCs.

**FIGURE 5:**
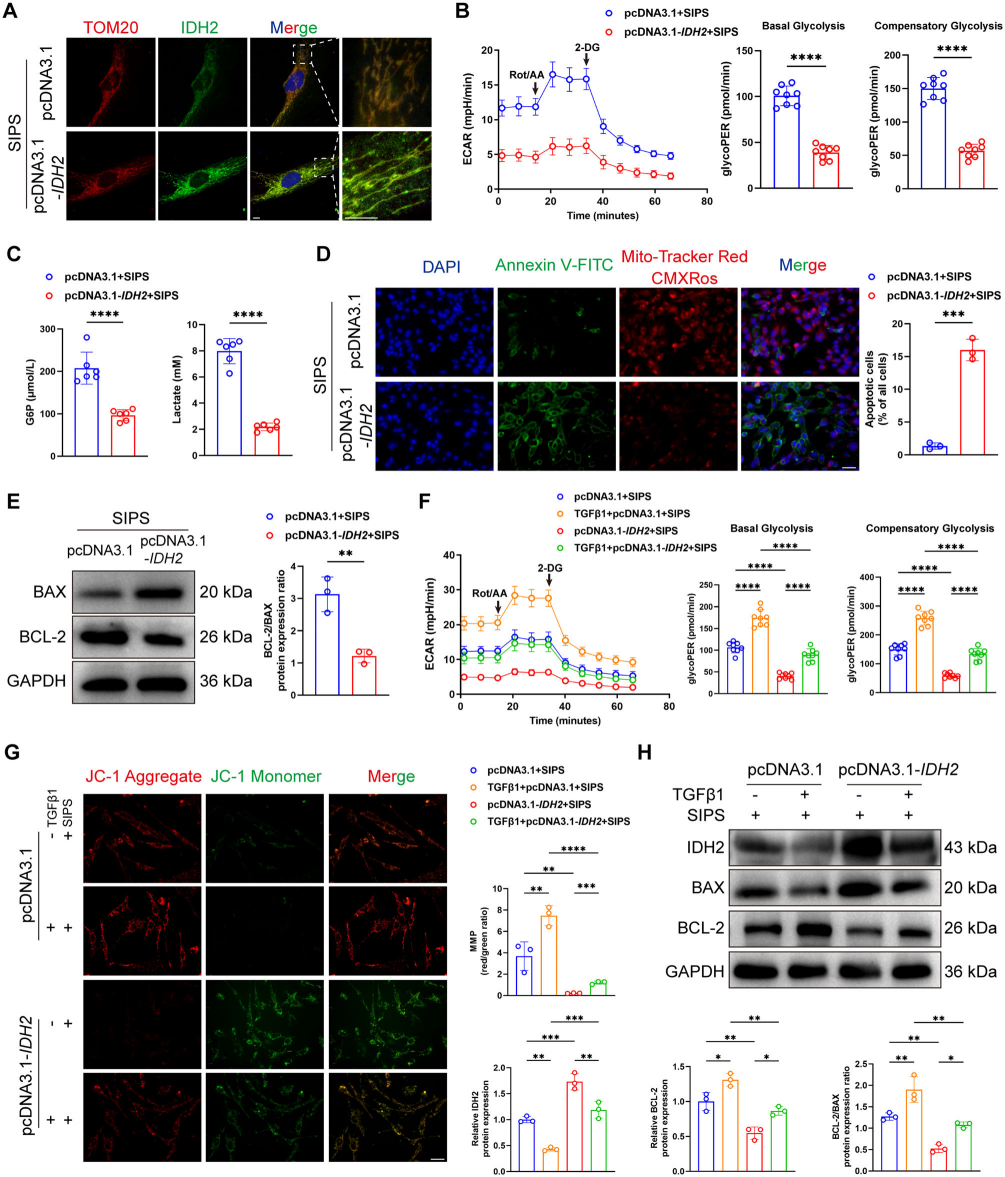
Overexpression of IDH2 reverses glycolysis and anti‐apoptotic phenotype in senescent VSMCs. (A) Representative immunofluorescence staining of TOM20 and IDH2 in senescent VSMCs transfected with pcDNA3.1 or pcDNA3.1‐*IDH2* (*n* = 3). Scale bar: 10 µm. (B) Seahorse XF analysis of ECAR and quantification of basal/compensatory glycolysis (*n* = 8). (C) G6P and lactate levels in IDH2‐overexpressing VSMCs in a SIPS state (*n* = 6). (D) Representative fluorescence images and quantitative analysis of apoptotic cells (Annexin V‐FITC, green) and mitochondrial tracking (Mito‐Tracker Red CMXRos, red) in VSMCs undergoing SIPS following IDH2 overexpression (*n* = 3). Scale bars: 50 µm. (E) Representative Western blot images and quantitative analysis of BCL‐2 and BAX expression in IDH2‐overexpressing senescent VSMCs (*n* = 3). (F) ECAR analysis and quantification of glycolysis in VSMCs undergoing SIPS transfected with IDH2 plasmids and treated with or without TGFβ1 (*n* = 8). (G) JC‐1 staining assessing MMP in the indicated VSMC groups (*n* = 3). Scale bars: 50 µm. (H) Representative Western blot images and quantitative analysis of IDH2, BAX, and BCL‐2 expression in VSMCs undergoing SIPS across the four designated treatment groups (*n* = 3). Data are presented as mean ± SD and were analyzed by two‐tailed unpaired Student's t‐test (A–E) or one‐way ANOVA followed by Tukey's multiple‐comparisons test (F–H). ^*^ *p*  <  0.05, ^**^ *p*  <  0.01, ^***^ *p*  <  0.001, ^****^ *p*  <  0.0001.

### 2.6 USP5 Directly Interacts With TGFBR1 and Inhibits its Ubiquitination

To elucidate the regulatory mechanism driving TGFBR1 accumulation, we first evaluated its transcriptional dynamics. Quantitative PCR revealed no significant difference in TGFBR1 mRNA levels between Ctrl cells and VSMCs in a SIPS state (Figure 6A), suggesting that TGFBR1 is primarily regulated at the post‐translational level. Utilizing the protein synthesis inhibitor cycloheximide (CHX), we observed that the half‐life of the TGFBR1 protein was significantly prolonged in VSMCs undergoing SIPS (Figure 6B). Crucially, treatment with the proteasome inhibitor MG132 forced the accumulation of TGFBR1 in Ctrl cells, abolishing the expression difference between the two groups (Figure 6C). These findings confirm that TGFBR1 stabilization during senescence is governed by the ubiquitin‐proteasome system. To identify the specific DUB responsible for this stabilization, we performed Co‐IP coupled with LC‐MS/MS. This analysis revealed enrichment of the deubiquitinases USP5 and USP12, with USP5 exhibiting a higher protein abundance and identification reliability (Figure 6D and Table S3). Co‐IP further validated that TGFBR1 directly interacted with USP5 and that this interaction was markedly enhanced in SIPS‐induced senescent VSMCs, whereas the USP12‐TGFBR1 interaction showed no obvious increase under SIPS conditions (Figure 6E and Figure S4A). Functionally, IP of TGFBR1 followed by immunoblotting for ubiquitin revealed a reduction in TGFBR1 ubiquitination in senescent VSMCs (Figure 6F). Immunofluorescence staining corroborated this by showing spatial colocalization of USP5 and TGFBR1, particularly accumulating at the cytoplasm and plasma membrane under SIPS conditions (Figure 6G). The scRNA‐seq analysis of atherosclerotic aortas revealed a significant positive correlation (R = 0.421, *p* < 0.001) between *Usp5* and *Tgfbr1* expression within the VSMC population (Figure 6H). Subsequently, we observed that in VSMCs with USP5 knockdown, the protein levels of TGFBR1 were significantly decreased, while the corresponding mRNA levels remained unchanged (Figure 6I,J). Moreover, USP12 knockdown did not significantly alter TGFBR1 protein expression in senescent VSMCs (Figure S4B). In vivo, USP5 knockdown did not induce compensatory upregulation of USP12 (Figure S4C), suggesting that USP12 is not the primary deubiquitinase responsible for TGFBR1 stabilization. CHX assays confirmed that USP5 deficiency accelerated the degradation and shortened the half‐life of TGFBR1 (Figure 6K). Notably, this downregulation of TGFBR1 was completely rescued by MG132 treatment (Figure 6L), suggesting that USP5 stabilizes TGFBR1 by antagonizing its proteasomal degradation. Consequently, USP5 knockdown disrupted the anti‐apoptotic phenotype, leading to a significant decrease in the BCL‐2/BAX ratio in VSMCs undergoing SIPS (Figure 6M). To complement these loss‐of‐function findings, we performed USP5 gain‐of‐function experiments in control VSMCs. Compared with cells transfected with an empty vector, USP5‐overexpressing VSMCs showed increased TGFBR1 expression, reduced IDH2 expression, enhanced G6P and lactate accumulation, and elevated p21 and p16 levels (Figure S5). Collectively, these data support USP5 as an important regulator of TGFBR1, stabilizing it to sustain the anti‐apoptotic senescent state.

**FIGURE 6:**
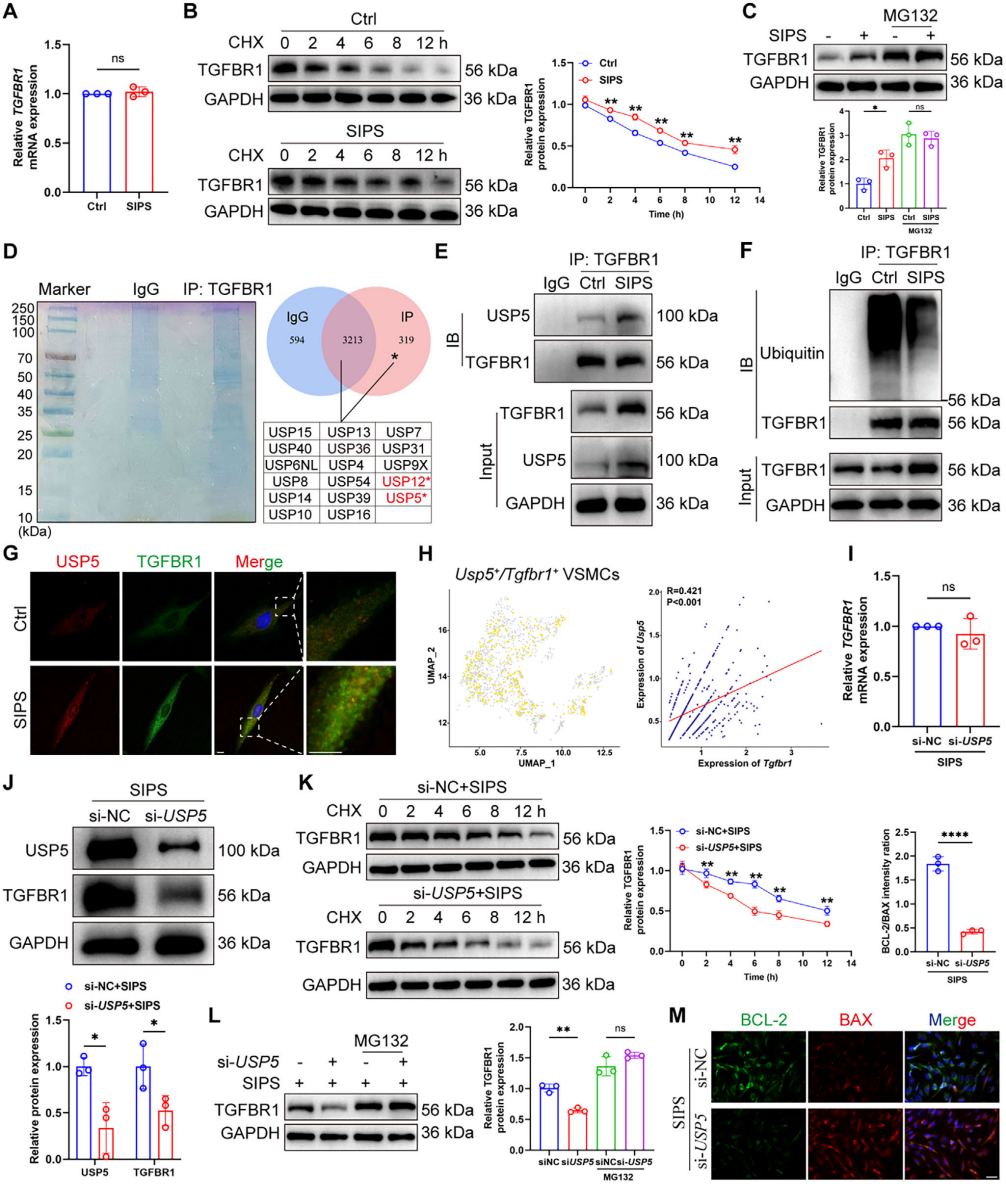
USP5 stabilizes TGFBR1 by suppressing ubiquitin‐proteasome degradation in senescent VSMCs. (A) Relative TGFBR1 mRNA expression in control VSMCs and VSMCs undergoing SIPS (*n* = 3). (B) Effect of cycloheximide (CHX, 10 µg/mL) chase on the degradation half‐life of TGFBR1 protein in control VSMCs and VSMCs in a SIPS state (*n* = 3). (C) Representative Western blot images and quantitative analysis of TGFBR1 expression in control VSMCs and VSMCs undergoing SIPS, treated with or without the proteasome inhibitor MG132 (*n* = 3). (D) Coomassie Brilliant Blue staining of the IgG or TGFBR1 immunoprecipitate, followed by LC‐MS/MS identification of enriched deubiquitinases. (E) Cell lysates from control VSMCs and VSMCs undergoing SIPS were subjected to Co‐immunoprecipitation (Co‐IP) with an anti‐TGFBR1 antibody or control IgG, followed by immunoblotting with anti‐USP5 and anti‐TGFBR1 antibodies. Whole‐cell lysates (Input) were simultaneously immunoblotted to verify protein expression levels (*n* = 3). (F) Control VSMCs and VSMCs in a SIPS state were treated with the proteasome inhibitor MG132 (50 µM) for 4 h. The cell lysates were then subjected to IP with an anti‐TGFBR1 antibody or control IgG, and subsequently immunoblotted with anti‐ubiquitin and anti‐TGFBR1 antibodies (*n* = 3). (G) Representative immunofluorescence images showing the colocalization of TGFBR1 (green) and USP5 (red) in VSMCs undergoing control and SIPS (*n* = 3). Scale bar: 10 µm. (H) UMAP plots and Pearson correlation analysis of *Usp5* and *Tgfbr*1 from scRNA‐seq data. (I) Relative TGFBR1 mRNA expression in VSMCs undergoing SIPS transfected with si‐NC or si‐*USP5* (*n* = 3). (J) Representative Western blot images and quantitative analysis of TGFBR1 and USP5 expression in VSMCs in a SIPS state following USP5 knockdown (*n* = 3). (K) Effect of CHX chase on the degradation half‐life of TGFBR1 protein in VSMCs undergoing SIPS transfected with si‐NC or si‐*USP5* (*n* = 3). (L) Representative Western blot images and quantitative analysis of TGFBR1 expression in si‐NC and si‐*USP5* VSMCs treated with MG132 under SIPS conditions, with quantification (*n* = 3). (M) Representative immunofluorescence images of BCL‐2 (green) and BAX (red) in senescent VSMCs following transfection with si‐NC or si‐*USP*5 (*n* = 3). Scale bar = 50 µm. Data are presented as mean ± SD and were analyzed by two‐tailed unpaired Student's t‐test (A, B, I–K, M), or one‐way ANOVA followed by Tukey's multiple‐comparisons test (C,L). ^*^ *p*  <  0.05, ^**^ *p*  <  0.01, ^****^ *p*  <  0.0001, ns: *p*  >  0.05.

### 2.7 USP5 Mediates K48‐Linked Deubiquitination of TGFBR1 at Lysine 213

To delineate the specific ubiquitin linkage targeted by USP5, cells were treated with MG132 to block proteasomal degradation and facilitate the detection of ubiquitination. In control VSMCs, TGFBR1 was predominantly modified by K48‐linked polyubiquitin chains. Notably, in VSMCs undergoing SIPS, the level of K48‐linked ubiquitination on TGFBR1 was markedly decreased, whereas K63‐linked modifications remained relatively unaltered (Figure 7A). Furthermore, knockdown of USP5 triggered a pronounced accumulation of K48‐linked ubiquitin chains on TGFBR1, without affecting K63‐linked polyubiquitin chains (Figure 7B). These data demonstrate that USP5 selectively removes K48‐linked polyubiquitin chains to protect TGFBR1 from degradation. To identify the specific ubiquitination sites, we utilized the GPS‐Uber online tool (http://gpsuber.biocuckoo.cn/online.php), which predicted eight potential ubiquitination sites within the TGFBR1 sequence (Figure 7C). Focusing on the highest‐scoring candidates, we generated three site‐directed mutants: TGFBR1‐K213R, K343R, and K449R. Co‐IP assays revealed that while the K343R and K449R mutants still exhibited deubiquitination under SIPS conditions identical to wild‐type (WT) TGFBR1, the K213R mutation largely abolished basal K48‐linked ubiquitination and rendered the protein unresponsive to these conditions (Figure 7D). Crucially, while USP5 knockdown increased the ubiquitination of WT, K343R, and K449R constructs, it completely failed to induce ubiquitin accumulation on the K213R mutant (Figure 7E). Structural modeling further indicated that K213 is located in a region favorable for interaction with USP5 (Figure 7F), supporting its role as a key regulatory residue. Finally, we assessed the functional consequences of this specific modification. Annexin V‐FITC staining demonstrated that expression of the TGFBR1‐K213R mutant markedly protected VSMCs in a SIPS state against apoptosis compared to WT‐expressing cells (Figure 7G). More importantly, while USP5 deficiency exacerbated apoptosis in WT cells, the K213R mutant was completely insensitive to USP5 deficiency, maintaining its anti‐apoptotic phenotype (Figure 7G). Consistently, SA‐β‐gal staining confirmed that the K213R mutant maintained a high senescence burden that could not be reversed by USP5 knockdown (Figure 7H), highlighting that K213‐mediated ubiquitination is essential for USP5 regulation of TGFBR1 stability and cellular senescence.

**FIGURE 7:**
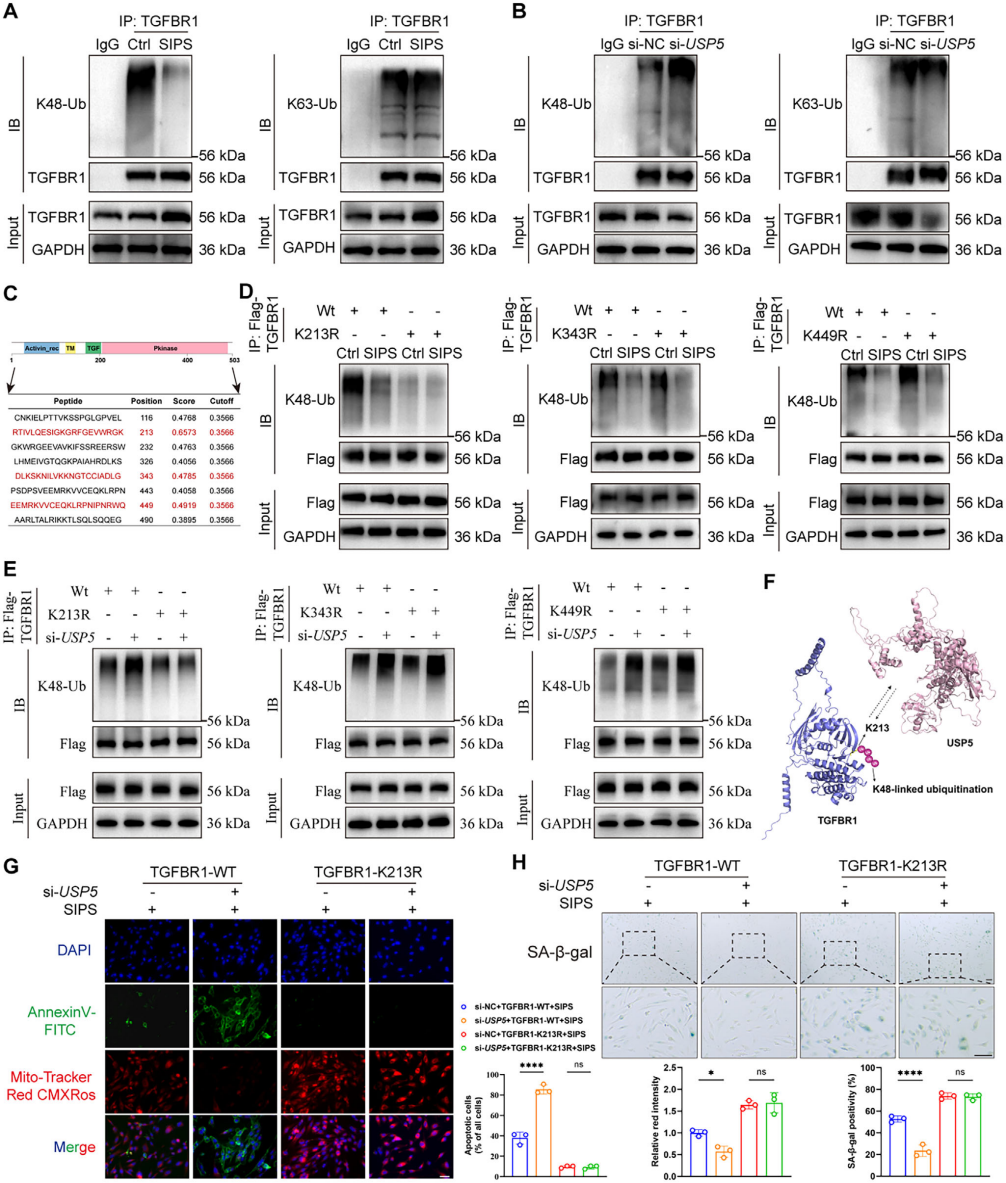
USP5 removes K48‐linked polyubiquitin chains from TGFBR1 at lysine 213. (A) Control VSMCs and VSMCs undergoing SIPS were treated with the proteasome inhibitor MG132 (50 µM) for 4 h. Cell lysates were then subjected to IP with anti‐TGFBR1 or anti‐IgG, followed by immunoblotting with anti‐K48‐linked ubiquitin, anti‐K63‐linked ubiquitin, and anti‐TGFBR1 antibodies (*n* = 3). (B) VSMCs in a SIPS state were transfected with si‐NC or si‐*USP5* and subsequently treated with MG132 (50 µM) for 4 h. The cell lysates were subjected to IP with anti‐TGFBR1 or anti‐IgG, and immunoblotted with the indicated antibodies (*n* = 3). (C) Bioinformatic prediction of potential ubiquitination sites on the TGFBR1 protein sequence utilizing the GPS‐Uber tool. (D) Control VSMCs and VSMCs undergoing SIPS were transfected with plasmids expressing Flag‐tagged wild‐type (WT) TGFBR1 or specific point mutants (K213R, K343R, K449R) and treated with MG132 (50 µM) for 4 h before harvest. Cell lysates were subjected to IP with anti‐Flag beads and immunoblotted with anti‐K48‐ubiquitin and anti‐Flag antibodies (*n* = 3). (E) VSMCs undergoing SIPS were co‐transfected with si‐NC or si‐*USP5* alongside the indicated Flag‐tagged WT or mutant TGFBR1 plasmids, and treated with MG132 (50 µM) for 4 h. The cell lysates were subjected to IP with anti‐Flag beads and immunoblotted with the indicated antibodies (*n* = 3). (F) Structural modeling of the interaction interface between USP5 (UniProt: P45974) and TGFBR1 (UniProt: P36897), highlighting the K213 ubiquitination site and the attached K48‐linked ubiquitin chain. (G) VSMCs in a SIPS state were co‐transfected with si‐NC or si‐*USP5* and Flag‐tagged WT or K213R TGFBR1 plasmids. Representative fluorescence images and quantitative analysis of apoptotic cells (Annexin V‐FITC, green) and mitochondrial tracking (Mito‐Tracker Red CMXRos, red) in VSMCs (*n* = 3). Scale bars = 50 µm. (H) SA‐β‐gal staining and quantification of VSMCs in a SIPS state from the indicated co‐transfection groups (*n* = 3). Scale bars = 100 µm. Data are presented as mean ± SD and were analyzed by one‐way ANOVA followed by Tukey's multiple‐comparisons test (G,H). ^*^ *p*  <  0.05, ^****^ *p*  <  0.0001, ns: *p*  >  0.05.

### 2.8 VSMC‐Specific USP5 Knockdown Reverses TGFBR1‐Driven Senescence and Atherosclerosis in Vivo

To investigate the functional interaction between USP5 and TGFBR1 in vivo, 8‐week‐old *Apoe* ^KO^ *Tgfbr1* ^SMCKI^ mice were intravenously injected with recombinant AAV9 vectors expressing a VSMC‐specific USP5 shRNA (AAV‐USP5) to knock down USP5, or a scrambled control sequence (AAV‐NC). *Apoe* ^KO^ *Tgfbr1* ^WT^ mice were administered AAV‐NC. Following AAV injection, all mice were fed a HFD for 12 weeks (Figure 8A). Immunofluorescence staining confirmed the specific downregulation of USP5 in VSMCs, validating targeted in vivo delivery (Figure S6). While body weight and serum lipid levels remained unchanged among groups (Figure 8B), targeted USP5 deficiency significantly suppressed the serum levels of pro‐inflammatory cytokines TNF‐α and IL‐6, which were elevated in the *Apoe* ^KO^ *Tgfbr1* ^SMCKI^ mice (Figure 8B). Under AAV‐NC treatment, *Apoe* ^KO^ *Tgfbr1* ^SMCKI^ mice exhibited a markedly aggravated atherosclerotic phenotype compared to *Apoe* ^KO^ *Tgfbr1* ^WT^ mice, characterized by expanded lesion burden and necrotic core areas, alongside massive lipid accumulation (Figure 8C). Crucially, immunofluorescence revealed an intense accumulation of TGFBR1 within senescent (p21^+^) VSMCs (α‐SMA^+^) in the plaques of *Apoe* ^KO^ *Tgfbr1* ^SMCKI^ mice. Notably, USP5 knockdown in vivo effectively abolished these pathological features, markedly reducing plaque size, lipid deposition, and the localized accumulation of TGFBR1 (Figure 8C). Mechanistically, USP5 deficiency disrupted the TGFBR1‐IDH2 signaling pathway in vivo. In the aortas of *Apoe* ^KO^ *Tgfbr1* ^SMCKI^ mice, targeted USP5 deficiency led to the degradation of TGFBR1 protein, which subsequently restored IDH2 expression (Figure 8D). This was accompanied by attenuation of glycolytic activity, as indicated by decreased G6P and lactate production (Figure 8E). Reduced glycolytic activity diminished apoptosis resistance in senescent VSMCs, as evidenced by a reduced BCL‐2/BAX ratio (Figure 8F). Consistently, a reduction in senescent cell accumulation was observed in the aortas, as evidenced by decreased SA‐β‐gal activity (Figure 8G) and lower expression of p21 and p16 (Figure 8H). Taken together, these data indicate that targeting USP5 disrupts the TGFBR1‐IDH2 axis, thereby reversing VSMC senescence and development of atherosclerosis.

**FIGURE 8:**
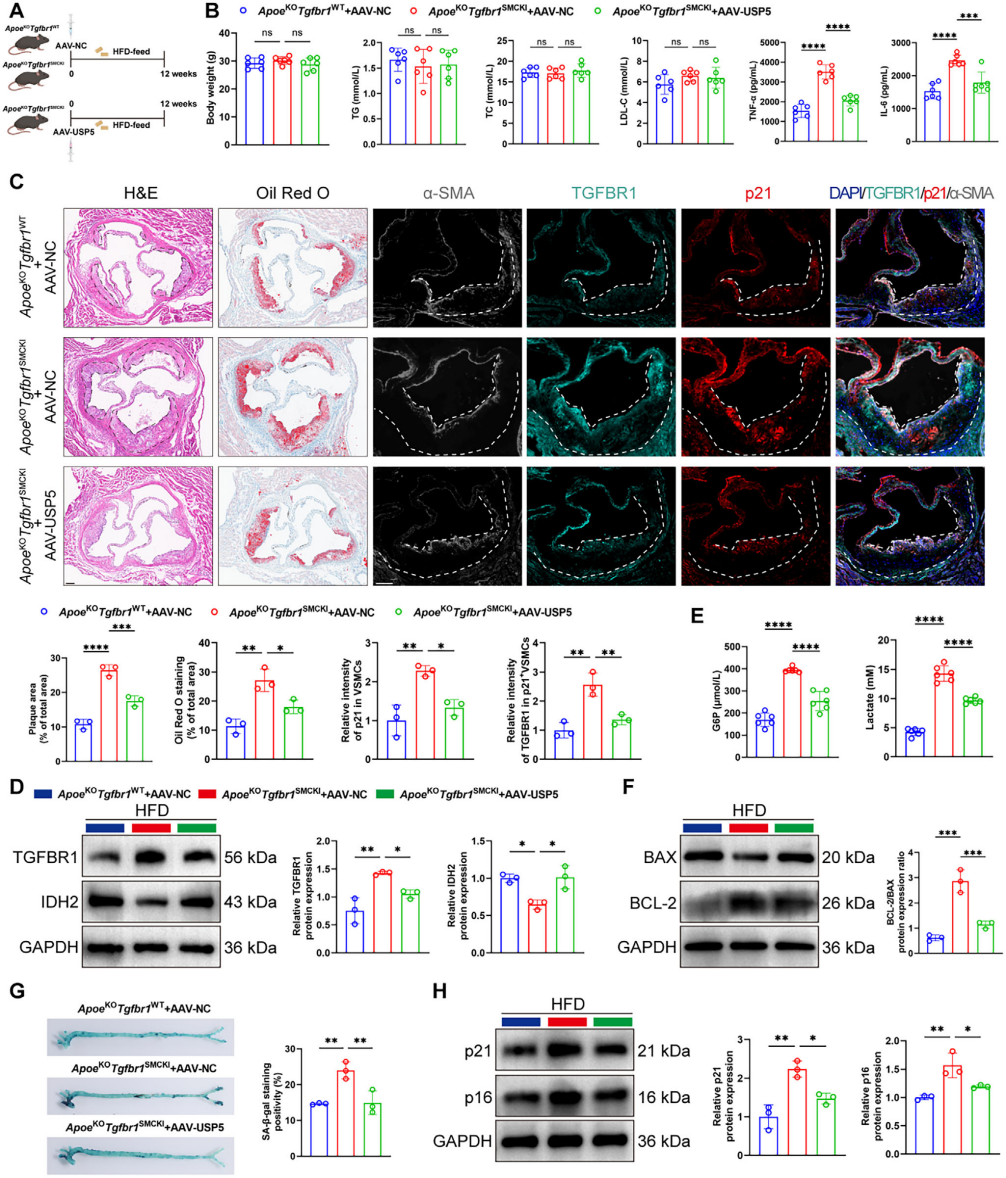
VSMC‐specific USP5 knockdown reverses VSMC senescence and atherosclerosis in *Apoe* ^KO^ *Tgfbr1* ^SMCKI^ mice. (A) Schematic of the in vivo experimental design. Eight‐week‐old *Apoe* ^KO^ *Tgfbr1* ^WT^ and *Apoe* ^KO^ *Tgfbr1* ^SMCKI^ mice were intravenously injected with recombinant AAVs expressing a VSMC‐specific USP5 shRNA (AAV‐USP5) or a scrambled control sequence (AAV‐NC), followed by 12 weeks of HFD feeding. (B) Body weight, serum triglyceride (TG), total cholesterol (TC), low‐density lipoprotein cholesterol (LDL‐C), tumor necrosis factor‐α (TNF‐α), and interleukin‐6 (IL‐6) levels in indicated groups (*n* = 6). (C) Representative cross‐sections of aortic root stained with H&E, Oil Red O, and immunofluorescence co‐staining for TGFBR1 (green), p21 (red), and α‐SMA (grey) (*n* = 3). Scale bar: 100 µm. The boundaries of the atherosclerotic lesions are demarcated by dashed lines. Accompanying bar graphs summarize the quantitative evaluations of total plaque area, Oil Red O‐positive lipid accumulation, and the corresponding immunofluorescence intensity within the plaque regions. (D) Representative Western blot images and quantitative analysis of TGFBR1 and IDH2 expression in mouse aortas (*n* = 3). (E) G6P and lactate concentrations measured in mouse aortic tissues (*n* = 6). (F) Representative Western blot images and quantitative analysis of BCL‐2 and BAX expression in mouse aortas (*n* = 3). (G) SA‐β‐gal staining of isolated mouse aortas with corresponding quantitative positivity rates (*n* = 3). (H) Representative Western blot images and quantitative analysis of p21 and p16 expression in mouse aortas (*n* = 3). Data are presented as mean ± SD and were analyzed by one‐way ANOVA followed by Tukey's multiple‐comparisons test (B–H). ^*^ *p*  <  0.05, ^**^ *p*  <  0.01, ^***^ *p*  <  0.001, ^****^ *p*  <  0.0001, ns: *p*  >  0.05.

## 3 Discussion

Cellular senescence in the vascular wall has been increasingly implicated in atherosclerotic plaque progression and instability [24, 25, 26]. Senescent VSMCs accumulate within atherosclerotic lesions, where they resist apoptosis and secrete pro‐inflammatory factors collectively known as the SASP, thereby promoting pathological vascular remodeling [27]. In this study, we identified a regulatory relationship linking TGFBR1 protein stability to metabolic changes in senescent VSMCs. We further identified the deubiquitinase USP5 as an upstream stabilizer of TGFBR1, which subsequently induces a metabolic reprogramming by reducing the expression of the mitochondrial enzyme IDH2. This USP5‐TGFBR1‐IDH2 axis maintains VSMCs in an apoptosis‐resistant, highly glycolytic senescent state, thereby promoting the progression of atherosclerosis (Figure 9).

**FIGURE 9:**
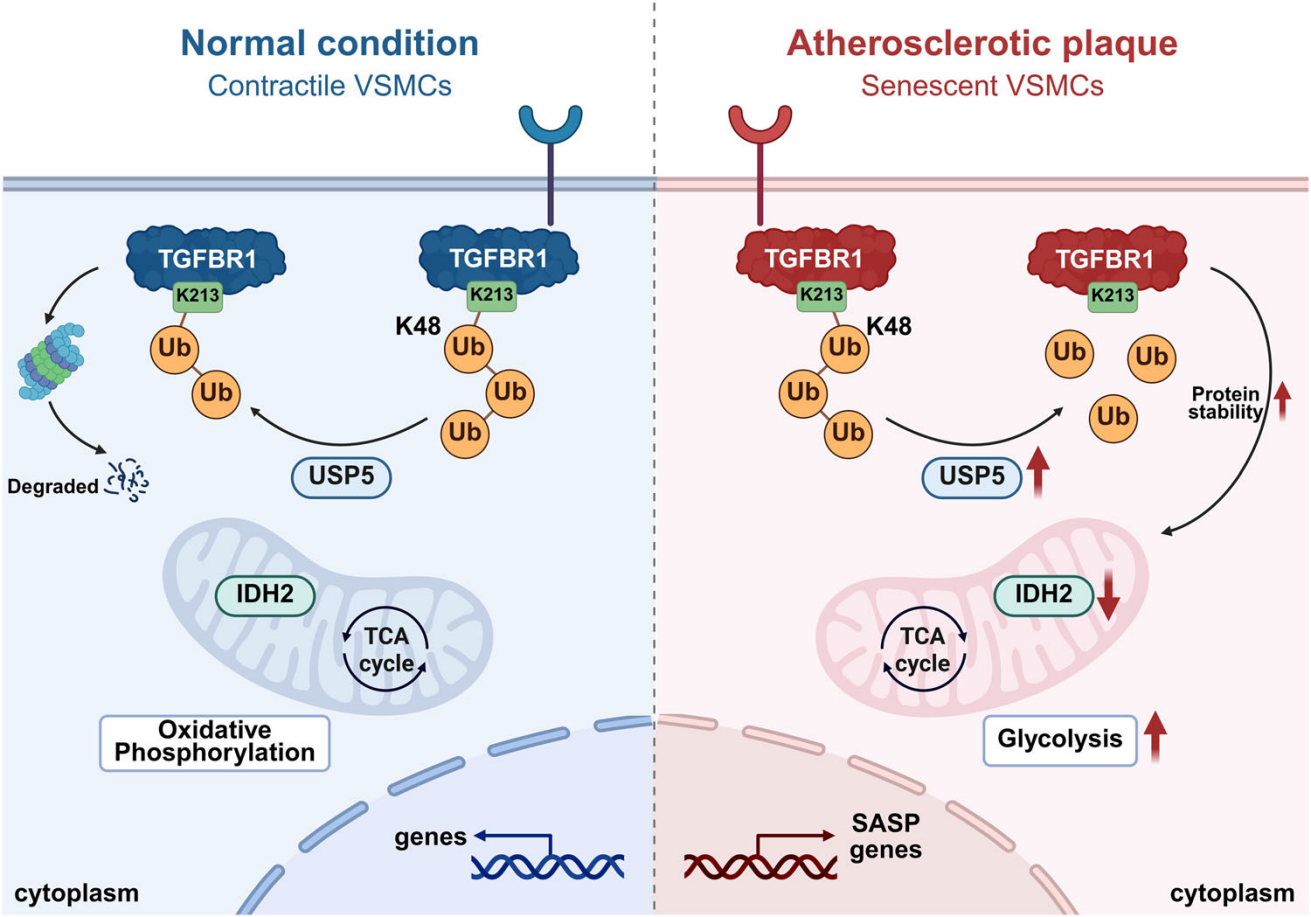
Schematic model illustrating the USP5‐TGFBR1‐IDH2 axis in driving VSMC senescence and atherosclerosis.

TGF‐β signaling in atherosclerosis has long been characterized by apparently conflicting observations [28, 29]. Early clinical and experimental studies have reported reduced circulating or vascular levels of TGF‐β in unstable plaques, supporting the concept that TGF‐β exerts anti‐inflammatory and plaque‐stabilizing effects [30, 31, 32]. More recent work, however, has revealed that TGF‐β signaling can actively promote atherosclerosis through multiple cellular mechanisms. One emerging mechanism involves endothelial‐to‐mesenchymal transition (EndMT), which is increasingly recognized as a common feature of human atherosclerotic lesions and contributes to endothelial dysfunction, plaque formation, and vascular remodeling [33]. TGF‐β signaling represents the most prominent inducer of this process, activating transcriptional programs that drive mesenchymal gene expression and structural remodeling of the vessel wall [34, 35]. In addition, endothelial TGF‐β signaling has been shown to directly promote vascular inflammation in hyperlipidemic models, where inhibition of endothelial TGF‐β signaling reduces vessel wall inflammation, vascular permeability, and disease progression [36]. Importantly, TGF‐β signaling also exerts profound effects on VSMCs, the predominant cellular component of advanced atherosclerotic plaques [37, 38]. VSMCs in diseased vessels frequently undergo phenotypic modulation from a contractile to a synthetic state, enabling them to proliferate, migrate, and remodel the extracellular matrix within the plaque [39, 40]. Premature cellular senescence further amplifies this process by driving stable phenotypic switching and rendering VSMCs resistant to re‐differentiation into the contractile phenotype, which may contribute to atherosclerosis and neointima formation [41]. Most prior studies have focused on ligand abundance, whereas the biological output of the pathway is also determined by receptor dynamics and signaling persistence. In this context, our findings shift attention toward receptor‐level regulation of TGF‐β signaling in VSMCs. Using scRNA‐seq analysis together with human plaque validation, we observed that TGFBR1 expression was markedly enriched in senescent VSMCs and positively correlated with the senescence marker p21, suggesting that receptor upregulation accompanies the emergence of the senescent phenotype in atherosclerotic lesions. Functional studies further demonstrated that VSMC‐specific TGFBR1 knockin significantly aggravated plaque development in *Apoe* ^KO^ mice, accompanied by enhanced senescence markers and SASP production. These results indicate that sustained activation of TGFBR1 signaling in VSMCs promotes a pro‐atherogenic cellular state characterized by stable senescence and resistance to apoptosis.

Proteomic analysis identified IDH2 as a downstream effector suppressed by sustained TGFBR1 signaling. As a core receptor kinase in canonical TGF‐β/SMAD signaling, activated TGFBR1 mediates SMAD2/3 phosphorylation [12]. Consistently, we observed increased phosphorylation of SMAD2 and SMAD3 in senescent VSMCs. However, SMAD3 knockdown failed to restore IDH2 expression in TGFBR1‐overexpressing senescent VSMCs, suggesting that TGFBR1‐mediated IDH2 suppression occurs through a SMAD‐independent mechanism. IDH2 is a mitochondrial NADP^+^‐dependent isocitrate dehydrogenase that catalyzes the conversion of isocitrate to α‐ketoglutarate within the tricarboxylic acid (TCA) cycle, thereby coupling mitochondrial oxidative metabolism with cellular redox homeostasis [42]. Emerging evidence suggests that dysregulation of IDH2 profoundly reshapes cellular metabolic programs. Previous studies have shown that inhibition of IDH2 activity disrupts mitochondrial metabolism and promotes a metabolic shift toward glycolysis, a phenomenon reminiscent of the Warburg effect observed in rapidly proliferating or stress‐adapted cells [43]. Consistent with this concept, a Wang et al. demonstrated that IDH2 deficiency alters TCA cycle flux and promotes glycolytic metabolism [23], indicating that IDH2 functions as a key regulator coordinating mitochondrial oxidative metabolism with cytosolic glucose utilization. These findings raise the possibility that IDH2 downregulation may provide a metabolic advantage for senescent cells, allowing them to maintain energy production and survival through glycolysis‐dependent metabolic reprogramming. In the present study, TGFBR1 overexpression markedly enhanced glycolytic activity, as evidenced by increased extracellular acidification rate and accumulation of glycolytic intermediates, whereas restoring IDH2 expression suppressed glycolysis and re‐sensitized senescent VSMCs to apoptosis. These results suggest that TGFBR1‐mediated repression of IDH2 drives a metabolic shift toward glycolysis, thereby sustaining the apoptosis‐resistant senescent phenotype of VSMCs. Our study provides mechanistic insight into how TGFBR1 sustains the persistence of senescent VSMCs.

While ubiquitin‐mediated degradation represents a mechanism for terminating TGF‐β signaling, the regulatory network controlling TGFBR1 stability is only partially understood [44]. Previous studies have demonstrated that SMAD7 recruits the HECT‐domain E3 ligase SMURF2 to the receptor complex, leading to ubiquitination and subsequent degradation of TGFBR1 [45]. To counterbalance this degradation process, several DUBs have been identified that stabilize TGFBR1 and sustain pathway activation. USP4 and USP15 have been previously identified as deubiquitinating enzymes that stabilize TGFBR1 in oncogenic contexts [46]. Notably, a recent study reported that the lysosomal membrane protein NPC1 directly interacts with TGFBR1 and prevents recruitment of the SMAD7‐SMURF1/2 complex, thereby reducing K48‐linked polyubiquitination and stabilizing the receptor [47]. However, the upstream regulatory mechanisms controlling TGFBR1 stability in vascular diseases remain largely unknown. In particular, whether a specific deubiquitinase governs TGFBR1 protein persistence during VSMC senescence has not been previously explored. In our study, we identify USP5 as a previously unrecognized stabilizer of TGFBR1 in atherosclerosis. Although USP12 was also detected in the TGFBR1 immunoprecipitate, it showed lower enrichment than USP5, and the USP12‐TGFBR1 association did not obviously increase under SIPS conditions. Together with the lack of significant effects of USP12 knockdown on TGFBR1 expression and the absence of compensatory USP12 upregulation after USP5 knockdown, these findings support USP5 as the predominant deubiquitinase regulating TGFBR1 stability in senescent VSMCs. Mechanistically, USP5 directly interacts with TGFBR1 and selectively removes K48‐linked polyubiquitin chains at lysine 213, thereby preventing proteasomal degradation and prolonging receptor stability. Unlike USP15, which stabilizes TGFBR1 indirectly through the SMAD7‐E3 ligase complex, or USP4, which regulates receptor trafficking in cancer cells, USP5 appears to function as a post‐translational checkpoint controlling TGFBR1 protein turnover in vascular cells. Our findings therefore expand the current understanding of TGFBR1 ubiquitination by identifying lysine 213 as a previously uncharacterized regulatory site involved in TGFBR1 ubiquitination. Notably, the scRNA‐seq analysis revealed a significant positive correlation between USP5 and TGFBR1 mRNA within the VSMC population of atherosclerotic aortas. This co‐expression pattern establishes an important spatial prerequisite for their functional interaction: USP5 and its substrate TGFBR1 are co‐produced within the same cell for post‐translational stabilization to occur. It should also be noted that, unlike the in vitro SIPS model in which TGFBR1 mRNA remained unchanged, *in* *vivo* scRNA‐seq data showed elevated TGFBR1 mRNA in VSMCs from atherosclerotic aortas. This difference likely reflects the cellular heterogeneity of the in vivo VSMC population, in which multiple phenotypic subsets may upregulate TGFBR1 transcription. Functionally, disruption of this deubiquitination axis markedly attenuated TGFBR1 signaling. USP5 deficiency promoted TGFBR1 degradation, restored IDH2 expression, suppressed glycolytic metabolism, and re‐sensitized senescent VSMCs to apoptosis both in vitro and in vivo. Sustained stabilization of TGFBR1 may therefore maintain persistent signaling in VSMCs, reinforcing the senescent phenotype and associated metabolic remodeling observed in atherosclerotic lesions.

From a translational perspective, the development of USP5 inhibitors may provide a promising pharmacological strategy for atherosclerosis. Recent studies have shown that USP5 is a druggable deubiquitinase. USP5 contains multiple ubiquitin‐binding modules, including the ZnF‐UBD, USP catalytic domain, and UBA domains, providing structural bases for small‐molecule intervention [48]. Structure‐activity relationship studies have identified ZnF‐UBD‐targeting USP5 ligands, such as compound 64, which binds USP5 with low‐micromolar affinity and inhibits USP5‐mediated cleavage of Lys48‐linked di‐ubiquitin in vitro [49]. Structure‐based virtual screening has further supported the feasibility of targeting the ZnF‐UBD of USP5 [50]. Moreover, the selective USP5 inhibitor WCY‐8‐67, which targets the UBA2 region, showed favorable in vivo bioavailability, tolerability, and therapeutic efficacy in leukemia xenograft models [51]. These findings support the pharmacological tractability of USP5. Future studies should therefore further validate existing USP5 inhibitors or develop more selective inhibitors, with a focus on assessing their therapeutic efficacy and long‐term cardiovascular safety in atherosclerosis.

Several limitations should be acknowledged. First, although our findings identify USP5 as a deubiquitinase that regulates TGFBR1 stability, the structural basis underlying substrate recognition remains to be defined. Second, our data support a SMAD‐independent, non‐canonical mechanism by which TGFBR1 promotes IDH2 downregulation and VSMC senescence. However, the precise downstream signaling events responsible for TGFBR1‐mediated IDH2 suppression remain to be further defined. Third, validation in larger human cohorts will be necessary to confirm clinical relevance. Future studies addressing these aspects will further refine the mechanistic framework proposed here.

## 4 Conclusion

Our findings reveal that USP5‐mediated deubiquitination stabilizes TGFBR1 and suppresses IDH2 expression, thereby promoting glycolytic reprogramming and sustaining apoptosis‐resistant senescence in VSMCs. This USP5‐TGFBR1‐IDH2 axis links post‐translational receptor stabilization with metabolic remodeling during atherosclerotic plaque progression. Targeting USP5‐dependent stabilization of TGFBR1 represents a potential strategy for limiting the accumulation of senescent VSMCs and attenuating atherosclerosis.

## 5 Experimental Methods

### 5.1 ScRNA‐seq Data Re‐Analysis

Publicly available scRNA‐seq data were obtained from the GEO database (accession number GSE239591), which includes aortic cells isolated from *p16‐tdTomato* ^+/−^ mice injected with AAV8‐PCSK9 and subsequently maintained on either NC or HFD for 16 weeks. Quality control, dimensionality reduction, and clustering were performed using Scanpy (v1.8.1) under Python 3.7. Cells were clustered using the Louvain algorithm, and clusters were visualized by Uniform Manifold Approximation and Projection (UMAP). For the comparison of differential genes between groups, the parameter was defined as log2|fold‐change|>0.25 and *p* < 0.05. Cell‐cell communication analysis was performed using CellChat (version 0.0.2) in R. Within the VSMC cluster, cells expressing *Cdkn1a* were classified as senescent VSMCs, and the remaining cells were classified as non‐senescent VSMCs. A CellChat object was generated using the normalized expression matrix and corresponding cell annotations. The ligand‐receptor interaction database was set, and the matching receptor inference calculation was performed.

### 5.2 Human Samples

Human carotid atherosclerotic plaque samples were collected from patients undergoing surgery at the Affiliated Hospital of Shandong University of Traditional Chinese Medicine. Clinical characteristics of the participants are summarized in Table S4. All procedures involving human tissues were performed in accordance with the Declaration of Helsinki and approved by the institutional ethics committee (Approval No. 2025‐034‐KY).

### 5.3 Animals

*Apoe* ^KO^ and *Tgfbr1* ^KI/WT^ mice were obtained from Vital River (Beijing, China) and GemPharmatech (Jiangsu, China), respectively. To generate VSMC‐specific TGFBR1 knockin mice, *Tgfbr1* ^KI/KI^ mice were crossed with Tagln‐Cre mice (GemPharmatech, China). *Apoe* ^KO^ *Tgfbr1* ^SMCKI^ mice were further generated by crossing *Tgfbr1* ^KI/KI^ Tagln‐Cre mice and *Apoe* ^KO^ mice. *Apoe* ^KO^ *Tgfbr1* ^WT^ mice served as the control group. Primer sequences used for genotyping are listed in Table S5. Atherosclerosis was induced in 8‐week‐old mice fed a NC or a HFD (21% fat and 0.5% cholesterol; KeaoXieli, Beijing, China) for 12 weeks. For in vivo silencing of USP5, we generated an adeno‐associated viral (AAV) vector driving the VSMC‐specific expression of a short hairpin RNA (shRNA) against USP5 under the control of the SM22α promoter (AAV‐USP5). A non‐targeting vector (AAV‐NC) was similarly constructed using the pAV‐sm22α‐GFP backbone. Viral vectors were administered via tail‐vein injection. All viral preparations were synthesized by Weizhen Biotechnology (Jinan, China). Following the interventions, animals were euthanized by inhalation of an overdose of isoflurane (5% in oxygen, administered once). Blood was then collected for serum preparation, and the heart and aorta were harvested for either fixation in 4% paraformaldehyde or storage at −80°C.

### 5.4 Cell Culture

VSMCs were cultured in Dulbecco's modified Eagle's medium supplemented with 10% fetal bovine serum and 1% penicillin‐streptomycin. Cells were maintained in a humidified incubator at 37°C with 5% CO_2_. To establish the SIPS model, VSMCs were exposed to 200 µM palmitate conjugated to 10% bovine serum albumin (PA‐BSA) for 48 h. Following this treatment, the PA‐BSA medium was replaced with standard complete medium for an additional 48 h to fully induce the senescent phenotype. The PA working solution was prepared by dissolving sodium palmitate (P9830, Solarbio) in sterile PBS at 70°C for 10 min, which was subsequently conjugated to fatty acid‐free BSA (A8850, Solarbio) at 50°C for 5 min. An equivalent concentration of BSA in PBS (10% BSA‐PBS) served as the vehicle control. Where indicated, cells were treated with the proteasome inhibitor MG132 (M126521, Aladdin), the protein synthesis inhibitor cycloheximide (CHX; HY‐13259, MedChemExpress), or recombinant TGFβ1 (HY‐P7118, MedChemExpress).

### 5.5 Statistical Analysis

All statistical analyses were performed using GraphPad Prism software (version 9.0, USA). Quantitative data are presented as mean ± standard deviation (SD). Data were first assessed for normality and homogeneity of variance. For comparisons between two independent groups, a two‐tailed unpaired Student's t‐test was used for normally distributed data, whereas the Mann‐Whitney U test was used for non‐normally distributed data. For comparisons among three or more groups, one‐way analysis of variance (ANOVA) followed by Tukey's multiple‐comparisons test was used for normally distributed data with equal variances, whereas the Kruskal‐Wallis test followed by Dunn's multiple‐comparisons test was used when these assumptions were not met. The specific statistical tests used for each analysis are indicated in the corresponding figure and table legends. A *p* value < 0.05 was considered statistically significant.

Additional methodological details are provided in the Supporting Information.

## Author Contributions

**Xinhai Cui**: Conceptualization, Data curation, Investigation, Methodology, Visualization, Writing – original draft. **Yuanlong Hu**: Investigation, Methodology, Visualization. **Lin Lin**: Investigation, Methodology. **Lei Zhang**: Investigation, Methodology. **Chao Li**: Conceptualization, Project administration, Funding acquisition, Supervision, Writing – review & editing. **Yunlun Li**: Project administration, Funding acquisition, Supervision, Writing – review & editing.

## Ethics Approval and Consent to Participate

All animal procedures were approved by the Ethics Committee of Shandong University of Traditional Chinese Medicine (Animal Ethics No. SDUTCM20230306001) and were conducted in accordance with the NIH Guide for the Care and Use of Laboratory Animals. Human samples were obtained with written informed consent, and the study was approved by the Ethics Committee of the Affiliated Hospital of Shandong University of Traditional Chinese Medicine (Approval No. 2025‐034‐KY) and conducted in accordance with the Declaration of Helsinki.

## Conflicts of Interest

The authors declare no conflicts of interest.

## Supporting information




**Supporting File**: advs76911‐sup‐0001‐SuppMat.docx.

## Data Availability

The data that support the findings of this study are available from the corresponding authors on reasonable request.
