# Supplementary material for: USP5 Stabilizes TGFBR1 to Drive Vascular Smooth Muscle Cell Senescence and Atherosclerosis
Source: Adv Sci (Weinh). 2026 Aug 5;13(60):e76911. doi: 10.1002/advs.76911 (PMC13440208; doi:10.1002/advs.76911)
Supplement: Supplementary file 1 [file ADVS-13-e76911-s001.docx]

***Supporting Information***

**USP5 Stabilizes TGFBR1 to Drive Vascular Smooth Muscle Cell Senescence and Atherosclerosis**

Xinhai Cui ^1^, Yuanlong Hu ^2^, Lin Lin ^2^, Lei Zhang ^3^, Chao Li ^1*^, and Yunlun Li ^2*^

*^1^**College of Traditional Chinese Medicine, Shandong University of Traditional Chinese Medicine, Jinan, 250355, China*

*^2^Innovation Research Institute of Traditional Chinese Medicine, Shandong University of Traditional Chinese Medicine, Jinan, 250355, China*

*^3^Department of Cardiovascular, Affiliated Hospital of Shandong University of Traditional Chinese Medicine, Jinan, 250014, China*

*Corresponding authors:

Chao Li at College of Traditional Chinese Medicine, Shandong University of Traditional Chinese Medicine, Jinan, 250355, China. E-mail: 60011986@sdutcm.edu.cn.

Yunlun Li at Innovation Research Institute of Traditional Chinese Medicine, Shandong University of Traditional Chinese Medicine, Jinan, 250355, China. E-mail: liyunlun0911@sdutcm.edu.cn.

**Figure S1. Senescent VSMCs exhibit enhanced predicted ligand-receptor communication with monocytes/macrophages.** CellChat analysis was performed on scRNA-seq data (GSE239591), and VSMCs were subdivided into senescent (*Cdkn1a*⁺) and non-senescent subpopulations. (**A**) Heatmap showing the number of inferred ligand-receptor interactions between senescent VSMCs, non-senescent VSMCs, and other major cell populations. (**B**) The communication strength between senescent VSMCs, non-senescent VSMCs, and other major cell populations. (**C**) Dot plot analysis of the specific ligand-receptor pairs mediating communication from senescent/non-senescent VSMCs to monocytes/macrophages (MPs). Dot color represents communication probability, and dot size indicates statistical significance.

**Figure S2.** **TGFBR1 suppresses IDH2 through a SMAD-independent pathway in senescent VSMCs.** (**A**) Representative Western blot images and quantitative analysis of TGFBR1, p-SMAD2/SMAD2, and p-SMAD3/SMAD3 expression in control VSMCs and VSMCs undergoing SIPS (n = 3). (**B**) SIPS-induced senescent VSMCs were co-transfected with negative control (si-NC) or SMAD3 knockdown (si-*SMAD3*) and either empty vector (pcDNA3.1) or TGFBR1 overexpression plasmid (pcDNA3.1-*TGFBR*1). Representative Western blot images and quantitative analysis of TGFBR1, SMAD3, IDH2, p21, and p16 in the indicated groups (n = 3). Data are presented as mean ± SD and were analyzed by two-tailed unpaired Student’s t-test (**A**) or one-way ANOVA followed by Tukey’s multiple-comparisons test (**B**). ^*^*P*  <  0.05, ^**^*P*  <  0.01, ns: *P*  >  0.05.

**Figure S3.** **Representative Western blot images and quantitative analysis of HK2, PFKFB3, and PKM2 expression in VSMCs undergoing SIPS transfected with IDH2 plasmids and treated with or without TGFβ1 (n = 3)**. Data are presented as mean ± SD and were analyzed by one-way ANOVA followed by Tukey’s multiple-comparisons test. ^*^*P*  <  0.05, ^**^*P*  <  0.01, ^***^*P*  <  0.001, ^****^*P*  <  0.0001.

**Figure S4.** **USP12 is not the major deubiquitinase responsible for TGFBR1 stabilization in senescent VSMCs.** (**A**) Cell lysates from control VSMCs and VSMCs undergoing SIPS were subjected to Co-immunoprecipitation (Co-IP) with an anti-TGFBR1 antibody or control IgG, followed by immunoblotting with anti-USP12 and anti-TGFBR1 antibodies. Whole-cell lysates (Input) were simultaneously immunoblotted to verify protein expression levels (n = 3). (**B**) Representative Western blot images and quantitative analysis of TGFBR1 and USP12 expression in VSMCs in a SIPS state following USP12 knockdown (n = 3). (**C**) Eight-week-old *Apoe*^KO^*Tgfbr1*^WT^ and *Apoe*^KO^*Tgfbr1*^SMCKI^ mice were intravenously injected with recombinant AAVs expressing a VSMC-specific USP5 shRNA (AAV-USP5) or a scrambled control sequence (AAV-NC), followed by 12 weeks of HFD feeding. Representative Western blot images and quantitative analysis of USP12 expression in mouse aortas (n = 3). Data are presented as mean ± SD and were analyzed by two-tailed unpaired Student’s t-test (**B**), or one-way ANOVA followed by Tukey’s multiple-comparisons test (**C**). ^**^*P*  <  0.01, ns: *P*  >  0.05.

**Figure S5.** **USP5 overexpression activates the TGFBR1-IDH2 axis and induces senescence in control VSMCs.** (**A**) Representative Western blot images and quantitative analysis of USP5, TGFBR1, IDH2, p21, and p16 expression in control VSMCs transfected with pcDNA3.1 or pcDNA3.1-*USP5* (n = 3). (**B**) G6P and lactate concentrations in control VSMCs following USP5 overexpression (n = 6). Data are presented as mean ± SD and were analyzed by two-tailed unpaired Student’s t-test. ^*^*P*  <  0.05, ^**^*P*  <  0.01, ^***^*P*  <  0.001.

**Figure S6.** Representative immunofluorescence images and quantitative analysis of USP5 (green) and α-SMA (red) colocalization in aortic root sections from *Apoe*^KO^*Tgfbr1*^WT^ mice injected with AAV-NC or AAV-USP5 (n = 3). Scale bar: 100 µm. Data are presented as mean ± SD and were analyzed by two-tailed unpaired Student’s t-test. ^***^*P*  <  0.001.

**Table S1.** **Detailed data shown in the heatmap in Figure 1J.**

| **SASP factor** | **Ctrl** | **RS** | **SIPS** | ***P* (Ctrl vs. RS)** | ***P* (RS vs. SIPS)** |
| --- | --- | --- | --- | --- | --- |
| TNF-α | 276.7 ± 73.06 | 753.1 ± 92.54 | 789.6 ± 192.8 | < 0.0001 | 0.8799 |
| IL-6 | 590.8 ± 133.2 | 1725 ± 162.1 | 1839 ± 123.1 | < 0.0001 | 0.3629 |
| IL-8 | 189.2 ± 65.78 | 1117 ± 75.34 | 1103 ± 116.2 | < 0.0001 | 0.9629 |
| IL-1β | 643.7 ± 85.29 | 1695 ± 89.20 | 1611 ± 75.46 | < 0.0001 | 0.2235 |
| CCL2 | 66.55 ± 10.58 | 223.1 ± 54.53 | 191.6 ± 73.27 | < 0.0001 | 0.5722 |

Data are presented as the mean ± SD (n = 6). *P* values were calculated using one-way ANOVA followed by Tukey’s multiple-comparisons test.

**Table S2.** **Detailed data shown in the heatmap in Figure 3G.**

| **SASP factor** | **pcDNA3.1+SIPS** | **pcDNA3.1-*TGFBR1***  **+SIPS** | ***P* (pcDNA3.1 vs. pcDNA3.1-*TGFBR1*)** |
| --- | --- | --- | --- |
| TNF-α | 855.6 ± 177.0 | 1150 ± 208.4 | 0.0250 |
| IL-6 | 1109 ± 189.2 | 2007 ± 451.2 | 0.0012 |
| IL-8 | 736.7 ± 222.8 | 1292 ± 178.7 | 0.0008 |
| IL-1β | 1216 ± 261.1 | 2221 ± 307.2 | 0.0001 |
| CCL2 | 203.3 ± 28.52 | 713.8 ± 89.06 | < 0.0001 |

Data are presented as the mean ± SD (n = 6). *P* values were calculated using a two-tailed unpaired Student’s t-test.

**Table S3.** **Co-IP/MS analysis of candidate TGFBR1-interacting deubiquitinases.**

| **Protein** | **Abundance** | **Unique peptides** | **Confidence score** |
| --- | --- | --- | --- |
| USP5 | 151.25 | 2 | 49.85 |
| USP12 | 43.38 | 1 | 37.05 |

**Table S4. Baseline data for patients.**

| **Sample** | **1** | **2** | **3** |
| --- | --- | --- | --- |
| Age | 75 | 61 | 63 |
| Sex | Male | Male | Male |
| Degree of stenosis | 70% | 90% | 95% |
| Symptom | No | No | Yes |

**Table S5. The primer sequences of genotyping.**

| **Primer name** | **Targeted sequences** |
| --- | --- |
| *Tgfbr1*-5’arm | GGGCAGTCTGGTACTTCCAAGCT (F) |
|  | TGGCGTTACTATGGGAACATACGTC (R) |
| *Tgfbr1*-3’arm | TTCAAGATCCGCCACAACATC(F) |
|  | ATATCCCCTTGTTCCCTTTCTGC (R) |
| *Tgfbr1*-WT | AGTCTTTCCCTTGCCTCTGCT(F) |
|  | GGGTCTTCCACCTTTCTTCAG (R) |
| *Tagln*-creERT2-5’arm | AGCAAGCAATGAGGGACAGGAG(F) |
|  | CATGTCCATCAGGTTCTTGCGAAC (R) |
| *Tagln*-creERT2-WT | TGAGGTTCAAGTTCAGAGGGGTC(F) |
|  | ATCCCTAGATTCCCTTCGCAAG (R) |
| *Apoe* | GCCTAGCCGAGGGAGAGCCG (Common) |
|  | GCCGCCCCGACTGCATCT (Mutant Reverse) |
|  | TGTGACTTGGGAGCTCTGCAGC (Wild type Reverse) |

**Table S6. The sequence of siRNAs.**

| **Gene name** | **Targeted sequences** |
| --- | --- |
| *USP5* | Sense: CCUGUCUGUAAGGACUUTT |
|  | Antisense: AAGUCUCCUUACAGACAGGTT |
| *SMAD3* | Sense: GGCCAACAUAGGCAAAUGATT |
|  | Antisense: UCAUUUGCCUAUGUUGGCCTT |
| *USP12* | Sense: GAAGAGAGAAAGCAGGAAATT |
|  | Antisense: UUUCCUGCUUUCUCUCUUCTT |

**Table S7. The primers sequence of RT-qPCR.**

| **Gene name** | **Targeted sequences** |
| --- | --- |
| *TGFBR1* | CAGCTTTGCCTGAACTCTCCT (F) |
|  | GACCCACATGGCTGTTTCCT (R) |
| *β-Actin* | CCTTCCTGGGCATGGAGTC (F) |
|  | TGATCTTCATTGTGCTGGGTG (R) |

**Table S8. The antibodies used for immunofluorescence staining.**

| **Antibody** | **Company** | **Catalog No.** | **Working Concentration** |
| --- | --- | --- | --- |
| TGFBR1 | Bioss | bs-0638R | 1:200 |
| p21 | Proteintech | 28248-1-AP | 1:200 |
| α-SMA | Proteintech | 14395-1-AP | 1:1600 |
| BCL-2 | Proteintech | 12789-1-AP | 1:200 |
| BAX | Proteintech | 50599-2-Ig | 1:200 |
| IDH2 | Proteintech | 15932-1-AP | 1:200 |
| TOM20 | Proteintech | 66777-1-Ig | 1:800 |
| USP5 | Proteintech | 66213-1-Ig | 1:400 |
| FITC conjugated Goat Anti-Rabbit IgG (H+L) | Servicebio | GB22303 | 1:200 |
| Cy3 conjugated Goat Anti-mouse IgG(H+L) | Servicebio | GB21301 | 1:200 |

**Table S9. The antibodies used for Western blot.**

| **Antibody** | **Company** | **Catalog No.** | **Working Concentration** |
| --- | --- | --- | --- |
| TGFBR1 | Abcam | ab235578 | 1:1000 |
| p21 | Proteintech | 82669-2-RR | 1:5000 |
| P16 | Cell Signaling Technology | 18769S | 1:1000 |
| BCL-2 | Proteintech | 12789-1-AP | 1:2000 |
| BAX | Proteintech | 50599-2-Ig | 1:50000 |
| IDH2 | Proteintech | 15932-1-AP | 1:1000 |
| USP5 | Proteintech | 66213-1-Ig | 1:2000 |
| Branched Ubiquitin Antibody Sampler Kit | Cell Signaling Technology | 33959 | 1:1000 |
| USP12 | ABclonal | A17862 | 1:1000 |
| HK2 | Cell Signaling Technology | 2867 | 1:1000 |
| PFKFB3 | Cell Signaling Technology | 13123 | 1:1000 |
| PKM2 | Cell Signaling Technology | 4053 | 1:1000 |
| p-SMAD2 | Cell Signaling Technology | 18338 | 1:1000 |
| SMAD2 | Cell Signaling Technology | 5339 | 1:1000 |
| p-SMAD3 | Cell Signaling Technology | 9520 | 1:1000 |
| SMAD3 | Cell Signaling Technology | 9523 | 1:1000 |
| GAPDH | Servicebio | ZB15004-HRP-100 | 1:1000 |
| HRP-conjugated goat anti-rabbit IgG (H+L) | ZSGB-BIO | ZB-2301 | 1:10000 |
| HRP-conjugated goat anti-mouse IgG (H+L) | ZSGB-BIO | ZB-2305 | 1:10000 |

**Experimental Methods**

**siRNA and plasmids** **transfection**

For transient knockdown of TGFBR1, USP5, USP12 or SMAD3, VSMCs were transfected with 10 nM of target-specific small interfering RNAs using the RNAFit transfection reagent (HB-RF-1000, HanBio) according to the manufacturer’s protocol. All siRNA sequences are detailed in **Table S6**.

For overexpression studies, plasmids encoding TGFBR1, IDH2 or USP5 were introduced into VSMCs using LipoFiter 3.0 transfection reagent (HB-LF3-1000, HanBio). To prepare the transfection complex, a certain amount of plasmid was diluted in a specific volume of serum-free medium, while a proportional volume of Lipofiter 3.0 reagent was mixed with an equal volume of medium. The diluted liposome was added dropwise to the plasmid solution, vortexed for 10 s, and incubated at room temperature for 20 min to allow stable complex formation. The complex was then added dropwise to the cell culture plate, gently mixed, and incubated at 37°C with 5% CO₂ for 3 h. All siRNA and plasmids were synthesized by HanBio (Shanghai, China).

**Total RNA extraction and real-time quantitative polymerase chain reaction (RT-qPCR)**

Total RNA was extracted from VSMCs using the SPARKeasy RNA extraction kit (AC0202, Sparkjade) according to the manufacturer’s instructions. Complementary DNA (cDNA) synthesis was performed using the SPARKscript II RT Plus kit (AG0304, Sparkjade). Real-time PCR amplification was carried out using SYBR Green Master Mix (AH0104, Sparkjade) on a Gentier 96E system (Tianlong, China). The primer sequences used are listed in **Table S7**. Relative gene expression levels were calculated using the 2^−ΔΔCT^ method with β-actin as the internal control.

**Immunofluorescence staining**

For cellular immunofluorescence, VSMCs were fixed with paraformaldehyde, permeabilized using Triton X-100, and blocked with bovine serum albumin to prevent nonspecific binding. Samples were incubated with primary antibodies overnight at 4°C and subsequently with fluorescence-labeled secondary antibodies. Nuclei were stained with DAPI. For tissue staining, aortic root cryosections underwent antigen retrieval and were blocked with 5% normal serum prior to overnight primary antibody incubation at 4°C. The sections were subsequently washed, probed with secondary antibodies, and mounted using a DAPI-containing medium for nuclear visualization. A comprehensive list of the antibodies utilized is provided in **Table S8**. Lipid droplets were visualized using BODIPY 493/503 (G1905, Servicebio) staining following the manufacturer’s protocol. Images were captured using a fluorescence microscope (ZEISS, Germany), and signal intensity was quantified with ImageJ software (NIH, USA).

**Immunohistochemical staining**

Paraffin-embedded tissue sections were deparaffinized, rehydrated, and subjected to antigen retrieval using EDTA buffer. Endogenous peroxidase activity was blocked with hydrogen peroxide, followed by incubation with 3% BSA. Sections were incubated with primary antibodie against CD68 (1:100 dilution, GB113109, Servicebio) overnight and then with HRP-conjugated goat anti-rabbit IgG antibody (ZB-2301, ZSGB-BIO). Signal detection was performed using DAB substrate, and nuclei were counterstained with hematoxylin.

**Western blot**

Protein lysates from cells or tissues were prepared using RIPA buffer (PC101, Epizyme) supplemented with protease and phosphatase inhibitors (1:100). After centrifugation, supernatants were collected and protein concentrations were determined using a BCA assay (ZJ102, Epizyme). Equal amounts of protein were separated by 10% SDS-PAGE and transferred to PVDF membranes (IPVH00010, Millipore). Membranes were blocked with 5% non-fat milk in TBST for 1 h at room temperature and incubated with primary antibodies overnight at 4°C. HRP-conjugated secondary antibodies were then applied. Protein signals were detected using an enhanced chemiluminescence system and quantified using ImageJ software. GAPDH was used as the loading control. Antibodies are detailed in **Table S9**.

**Co-immunoprecipitation (Co-IP) assay and LC-MS/MS analysis**

Cell lysates were incubated with Protein A/G magnetic beads (P2179M, Beyotime) conjugated with antibodies against TGFBR1 or control IgG. After overnight incubation, the immunoprecipitated complexes were washed and eluted with SDS-PAGE loading buffer by heating at 95°C for 5 min. Additionally, Input and immunoprecipitated samples were analyzed by Western blot using antibodies against TGFBR1, USP5, and USP12. Protein samples from excised gel bands were subjected to in-gel digestion, and the resulting peptides were analyzed by LC-MS/MS (Novogene, China) to identify deubiquitinating enzymes interacting with TGFBR1.

**Ubiquitination analysis**

VSMCs were treated with 50 μM of the proteasome inhibitor MG132 for 4 h. Protein extracts were immunoprecipitated using anti-TGFBR1 antibodies. The ubiquitination status of TGFBR1 was evaluated by immunoblotting using antibodies recognizing total ubiquitin, K48-linked ubiquitin, or K63-linked ubiquitin chains. For site-specific analysis, VSMCs were transfected with plasmids expressing Flag wild-type (WT) or mutant TGFBR1 (Flag-K213R, Flag-K343R, or Flag-K449R). VSMCs were lysed in buffer containing 1% SDS, boiled to denature proteins, and subsequently diluted to a final SDS concentration of 0.1%. IP and Western blot were performed to determine ubiquitination levels.

**Determination of serum lipid concentration**

Serum was centrifuged at 1,301 × g for 10 min at 4°C, and the levels of total cholesterol (TC), triglyceride (TG), and low-density lipoprotein cholesterol (LDL-C) were determined using an automatic biochemistry analyzer (Kehua Bio-Engineering, China).

**Atherosclerotic lesion analysis**

Hearts were embedded in optimal cutting temperature compound and cryosectioned at an 8-μm thickness. Sections were stained with Oil Red O for 15 min to visualize lipid deposition, differentiated in 75% ethanol, and counterstained with hematoxylin. Hematoxylin and eosin (H&E) and Masson staining were additionally performed according to standard protocols (Servicebio). For en face analysis, fixed whole aortas were meticulously cleared of adventitial tissue, opened longitudinally, and stained with a 0.5% Oil Red O solution. Images were captured using a light microscope (Leica, Germany), and the atherosclerotic burden was quantified as the percentage of the lesion area relative to the total surface area.

**Senescence-associated beta galactosidase (SA-β-gal) staining**

Cellular senescence was evaluated using a senescence-associated β-galactosidase staining kit (C0602, Beyotime). Cells were fixed and incubated with staining solution at 37°C overnight. For mouse aortas, after removal of the surrounding adipose tissue, the staining was performed using the same procedure as described above. Images were obtained using a microscope (ZEISS, Germany). For quantification of SA-β-gal staining, images were analyzed using ImageJ software. All images were coded before analysis, and quantification was performed by an investigator blinded to the experimental groups. Blue SA-β-gal-positive areas were manually delineated according to consistent staining criteria across all samples. SA-β-gal positivity was calculated as the positive stained area divided by the total aortic area and expressed as a percentage.

**Enzyme-linked immunosorbent assay (ELISA)**

Commercially available ELISA kits (Jianglaibio, China) were used to measure the levels of tumor necrosis factor-α (TNF-α), interleukin-6 (IL-6), interleukin-8 (IL-8), interleukin-1β (IL-1β), and C-C motif chemokine ligand 2 (CCL2) in cell culture supernatants and serum samples, strictly following the manufacturer's instructions.

**Detection of mitochondrial membrane potential (MMP)** **and cell apoptosis**

VSMCs were incubated with JC-1 working solution (C2006, Beyotime) at 37°C for 20-30 min. After incubation, cells were washed twice with pre-cooled JC-1 staining buffer to remove unbound dye. Fluorescence was then detected using a fluorescence microscope. The ratio of red (aggregates, excitation/emission = 525/590 nm) to green (monomers, excitation/emission = 490/530 nm) fluorescence intensity was calculated to reflect changes in MMP. Apoptosis was determined using an Annexin V-FITC apoptosis detection kit (C1071S, Beyotime) following the recommended protocol.

**Proteomic analysis**

Aortic tissue samples (n = 3 per group) were collected from *Apoe*^KO^*Tgfbr1*^WT^ and *Apoe*^KO^*Tgfbr1*^SMCKI^ mice following 12 weeks of HFD feeding, and subjected to proteomic analysis by Majorbio Bio-pharm Technology Co., Ltd. (Shanghai, China). Total protein was extracted using a lysis buffer supplemented with protease inhibitors. The protein lysates were sequentially reduced, alkylated, and digested with trypsin. The resulting peptides were desalted, quantified, and analyzed via LC-MS/MS. Acquired MS/MS spectra were searched against the appropriate Mus musculus protein database. Differentially expressed proteins (DEPs) between the two groups were identified using a threshold of |log₂ fold change| ≥ 0.5 and *P* < 0.05. Subsequent bioinformatics evaluations, including functional annotation, Gene Set Enrichment Analysis (GSEA), and KEGG pathway enrichment, were executed utilizing the Majorbio Cloud platform (www.majorbio.com).

**Seahorse assay**

Glycolytic rate was assessed using the Seahorse XFe96 Analyzer (Agilent, USA). VSMCs were resuspended in Seahorse XF Assay Medium and seeded into an Agilent Seahorse XFe96/XF Pro Cell Culture Microplate at a density of 5,000 cells per well. The Extracellular Acidification Rate (ECAR) was recorded using the Seahorse XF Glycolytic Rate Assay Kit (103344-100, Agilent). The following reagents were sequentially added to modulate ECAR: rotenone/antimycin A (Rot/AA, 0.5 μM) and 2-Deoxy-D-glucose (2-DG, 50 mM). The glycolytic rate was calculated according to the manufacturer’s instructions.

**Measurement of glucose-6-phosphate (G6P) and lactate levels**

Intracellular G6P levels were determined using a commercial detection kit (S0185, Beyotime) according to the manufacturer’s instructions. Briefly, VSMCs were harvested, washed with cold PBS, and lysed in the extraction buffer provided in the kit. Cell lysates were centrifuged to obtain clarified supernatants, which were subsequently incubated with the detection reagent. Absorbance at 450 nm was measured, and G6P levels were calculated based on the standard curve. Lactate levels in the culture medium were measured using a Lactic Acid Detection Kit (S0208S, Beyotime) following the manufacturer’s protocol. For animal samples, aortic tissues were homogenized, and G6P and lactate levels in the resulting tissue homogenates were determined.

**Un-cropped images presented in manuscript**

**The lanes corresponding to the cropped images in the figures are indicated by red boxes.**

**Figure 1G**

| TGFBR1 |  |  |  |
| --- | --- | --- | --- |
| p21 |  |  |  |
| p16 |  |  |  |
| GAPDH |  |  |  |

**Figure 1I**

| TGFBR1 |  |  |  |
| --- | --- | --- | --- |
| p21 |  |  |  |
| p16 |  |  |  |
| GAPDH |  |  |  |

**Figure 2A**

| TGFBR1 |  |  |  |
| --- | --- | --- | --- |
| GAPDH |  |  |  |

**Figure 2J**

| TGFBR1 |  |  |  |
| --- | --- | --- | --- |
| p21 |  |  |  |
| p16 |  |  |  |
| GAPDH |  |  |  |

**Figure 3B**

| BAX |  |  |  |
| --- | --- | --- | --- |
| BCL-2 |  |  |  |
| GAPDH |  |  |  |

**Figure 3D**

| TGFBR1 |  |  |  |
| --- | --- | --- | --- |
| GAPDH |  |  |  |
| BAX |  |  |  |
| BCL-2 |  |  |  |
| GAPDH |  |  |  |

**Figure 3H**

| p21 |  |  |  |
| --- | --- | --- | --- |
| p16 |  |  |  |
| GAPDH |  |  |  |

**Figure 4K**

| TGFBR1 |  |  |  |
| --- | --- | --- | --- |
| GAPDH |  |  |  |
| IDH2 |  |  |  |
| GAPDH |  |  |  |

**Figure 5E**

| BAX |  |  |  |
| --- | --- | --- | --- |
| GAPDH |  |  |  |
| BCL-2 |  |  |  |
| GAPDH |  |  |  |

**Figure 5H**

| IDH2 |  |  |  |
| --- | --- | --- | --- |
| GAPDH |  |  |  |
| BAX |  |  |  |
| BCL-2 |  |  |  |
| GAPDH |  |  |  |

**Figure 6B**

| Ctrl |  |  |  |
| --- | --- | --- | --- |
| TGFBR1 |  |  |  |
| GAPDH |  |  |  |
| SIPS |  |  |  |
| TGFBR1 |  |  |  |
| GAPDH |  |  |  |

**Figure 6C**

| TGFBR1 |  |  |  |
| --- | --- | --- | --- |
| GAPDH |  |  |  |

**Figure 6E**

| IB |  |  |  |
| --- | --- | --- | --- |
| USP5 |  |  |  |
| TGFBR1 |  |  |  |
| Input |  |  |  |
| TGFBR1 |  |  |  |
| GAPDH |  |  |  |
| USP5 |  |  |  |
| GAPDH |  |  |  |

**Figure 6F**

| IB |  |  |  |
| --- | --- | --- | --- |
| Ubiquitin |  |  |  |
| TGFBR1 |  |  |  |
| Input |  |  |  |
| TGFBR1 |  |  |  |
| GAPDH |  |  |  |

**Figure 6J**

| USP5 |  |  |  |
| --- | --- | --- | --- |
| GAPDH |  |  |  |
| TGFBR1 |  |  |  |
| GAPDH |  |  |  |

**Figure 6K**

| si-NC+SIPS |  |  |  |
| --- | --- | --- | --- |
| TGFBR1 |  |  |  |
| GAPDH |  |  |  |
| si-*USP5*+SIPS |  |  |  |
| TGFBR1 |  |  |  |
| GAPDH |  |  |  |

**Figure 6L**

| TGFBR1 |  |  |  |
| --- | --- | --- | --- |
| GAPDH |  |  |  |

**Figure 7A**

| IB |  |  |  |
| --- | --- | --- | --- |
| K48-Ub |  |  |  |
| TGFBR1 |  |  |  |
| Input |  |  |  |
| TGFBR1 |  |  |  |
| GAPDH |  |  |  |
|  |  |  |  |
| IB |  |  |  |
| K63-Ub |  |  |  |
| TGFBR1 |  |  |  |
| Input |  |  |  |
| TGFBR1 |  |  |  |
| GAPDH |  |  |  |

**Figure 7B**

| IB |  |  |  |
| --- | --- | --- | --- |
| K48-Ub |  |  |  |
| TGFBR1 |  |  |  |
| Input |  |  |  |
| TGFBR1 |  |  |  |
| GAPDH |  |  |  |
|  |  |  |  |
| IB |  |  |  |
| K63-Ub |  |  |  |
| TGFBR1 |  |  |  |
| Input |  |  |  |
| TGFBR1 |  |  |  |
| GAPDH |  |  |  |

**Figure 7D**

| K213R:  IB |  |  |  |
| --- | --- | --- | --- |
| K48-Ub |  |  |  |
| Flag |  |  |  |
| Input |  |  |  |
| Flag |  |  |  |
| GAPDH |  |  |  |
|  |  |  |  |
| K343R:  IB |  |  |  |
| K48-Ub |  |  |  |
| Flag |  |  |  |
| Input |  |  |  |
| Flag |  |  |  |
| GAPDH |  |  |  |
|  |  |  |  |
| K449R:  IB |  |  |  |
| K48-Ub |  |  |  |
| Flag |  |  |  |
| Input |  |  |  |
| Flag |  |  |  |
| GAPDH |  |  |  |

**Figure 7E**

| K213R:  IB |  |  |  |
| --- | --- | --- | --- |
| K48-Ub |  |  |  |
| Flag |  |  |  |
| Input |  |  |  |
| Flag |  |  |  |
| GAPDH |  |  |  |
|  |  |  |  |
| K343R:  IB |  |  |  |
| K48-Ub |  |  |  |
| Flag |  |  |  |
| Input |  |  |  |
| Flag |  |  |  |
| GAPDH |  |  |  |
|  |  |  |  |
| K449R:  IB |  |  |  |
| K48-Ub |  |  |  |
| Flag |  |  |  |
| Input |  |  |  |
| Flag |  |  |  |
| GAPDH |  |  |  |

**Figure 8D**

| TGFBR1 |  |  |  |
| --- | --- | --- | --- |
| GAPDH |  |  |  |
| IDH2 |  |  |  |
| GAPDH |  |  |  |

**Figure 8F**

| BAX |  |  |  |
| --- | --- | --- | --- |
| GAPDH |  |  |  |
| BCL-2 |  |  |  |
| GAPDH |  |  |  |

**Figure 8H**

| p21 |  |  |  |
| --- | --- | --- | --- |
| GAPDH |  |  |  |
| p16 |  |  |  |
| GAPDH |  |  |  |

**Figure S2A**

| TGFBR1 |  |  |  |
| --- | --- | --- | --- |
| GAPDH |  |  |  |
| P-SMAD2 |  |  |  |
| GAPDH |  |  |  |
| SMAD2 |  |  |  |
| GAPDH |  |  |  |
| P-SMAD3 |  |  |  |
| GAPDH |  |  |  |
| SMAD3 |  |  |  |
| GAPDH |  |  |  |

**Figure S2B**

| TGFBR1 |  |  |  |
| --- | --- | --- | --- |
| GAPDH |  |  |  |
| SMAD3 |  |  |  |
| GAPDH |  |  |  |
| IDH2 |  |  |  |
| p21 |  |  |  |
| GAPDH |  |  |  |
| P16 |  |  |  |
| GAPDH |  |  |  |

**Figure S3**

| HK2 |  |  |  |
| --- | --- | --- | --- |
| GAPDH |  |  |  |
| PFKFB3 |  |  |  |
| GAPDH |  |  |  |
| PKM2 |  |  |  |
| GAPDH |  |  |  |

**Figure S4A**

| IB |  |  |  |
| --- | --- | --- | --- |
| USP12 |  |  |  |
| TGFBR1 |  |  |  |
| Input |  |  |  |
| TGFBR1 |  |  |  |
| USP12 |  |  |  |
| GAPDH |  |  |  |

**Figure S4B**

| USP12 |  |  |  |
| --- | --- | --- | --- |
| TGFBR1 |  |  |  |
| GAPDH |  |  |  |

**Figure S4C**

| USP12 |  |  |  |
| --- | --- | --- | --- |
| GAPDH |  |  |  |

**Figure S5A**

| USP5 |  |  |  |
| --- | --- | --- | --- |
| TGFBR1 |  |  |  |
| GAPDH |  |  |  |
| IDH2 |  |  |  |
| p21 |  |  |  |
| GAPDH |  |  |  |
| p16 |  |  |  |
| GAPDH |  |  |  |
